# Evaluation of genome and base editing tools in maize protoplasts

**DOI:** 10.3389/fpls.2022.1010030

**Published:** 2022-11-28

**Authors:** Yannick Fierlej, Nathanaël M. A. Jacquier, Loïc Guille, Jérémy Just, Emilie Montes, Christelle Richard, Jeanne Loue-Manifel, Nathalie Depège-Fargeix, Antoine Gaillard, Thomas Widiez, Peter M. Rogowsky

**Affiliations:** ^1^ Laboratoire Reproduction et Développement des Plantes, Univ Lyon, Ecole Normale Supérieure (ENS) de Lyon, Université Claude Bernard (UCB) Lyon 1, Centre National de la Recherche Scientifique (CNRS), Institut National de Recherche pour l'Agriculture, l'alimentation et l'Environnement (INRAE), Lyon, France; ^2^ Department Research and Development, MAS Seeds, Haut-Mauco, France

**Keywords:** genome editing, plant biotechnology, protoplast, sgRNA scaffold, stomatal development, targeted mutagenesis, CRISPR/Cas9, *Zea mays*

## Abstract

**Introduction:**

Despite its rapid worldwide adoption as an efficient mutagenesis tool, plant genome editing remains a labor-intensive process requiring often several months of *in vitro* culture to obtain mutant plantlets. To avoid a waste in time and money and to test, in only a few days, the efficiency of molecular constructs or novel Cas9 variants (clustered regularly interspaced short palindromic repeats (CRISPR)-associated protein 9) prior to stable transformation, rapid analysis tools are helpful.

**Methods:**

To this end, a streamlined maize protoplast system for transient expression of CRISPR/Cas9 tools coupled to NGS (next generation sequencing) analysis and a novel bioinformatics pipeline was established.

**Results and discussion:**

Mutation types found with high frequency in maize leaf protoplasts had a trend to be the ones observed after stable transformation of immature maize embryos. The protoplast system also allowed to conclude that modifications of the sgRNA (single guide RNA) scaffold leave little room for improvement, that relaxed PAM (protospacer adjacent motif) sites increase the choice of target sites for genome editing, albeit with decreased frequency, and that efficient base editing in maize could be achieved for certain but not all target sites. Phenotypic analysis of base edited mutant maize plants demonstrated that the introduction of a stop codon but not the mutation of a serine predicted to be phosphorylated in the bHLH (basic helix loop helix) transcription factor ZmICEa (INDUCER OF CBF EXPRESSIONa) caused abnormal stomata, pale leaves and eventual plant death two months after sowing.

## Introduction

Genome editing using clustered regularly interspaced short palindromic repeats (CRISPR)/CRISPR-associated protein 9 (Cas9) technology has rapidly become the preferred tool to generate mutants for functional genomics in microbes, animals and plants (Adli, 2018; Chen et al., 2019; Schindele et al., 2020). The success of CRISPR/Cas9 technology over earlier meganuclease, zinc finger or transcription activator-like effector nuclease (TALEN) techniques is mainly due to the fact that the recognition of the target sequence in the genome is mediated by fully foreseeable DNA/RNA base pairing rather than less predictable DNA/protein interactions. In its original context of bacterial defense the Cas9 nuclease forms a complex with two RNA molecules, the CRISPR RNA (crRNA) and the trans-activating crRNA (tracrRNA) (Jinek et al., 2012), which for biotechnological applications were linked together into a single-guide RNA (sgRNA) (Mali et al., 2013). Cas9 expression is driven either by constitutive or tissue-specific promoters transcribed by RNA polymerase II, whereas the sgRNA is generally under the control of U3 or U6 promoters transcribed by RNA polymerase III.

In plants, the most widely use of the technology is targeted mutagenesis, which is achieved by a CRISPR/Cas9-mediated double strand break of the DNA. Due to the random nature of error-prone cellular DNA repair, only the site but not the nature of the mutation is predetermined. In 2019, 97% of the publications were based on this approach and only 3% used true genome editing, which copies the modified, predetermined sequence of a repair matrix into the genome (Modrzejewski et al., 2019). This preference is due to the fact that the molecular nature of the mutation is not crucial for the generation of loss-of-function mutants and that the repair of nuclease-mediated double strand breaks by non-homologous end joining (NHEJ) or microhomology-mediated end joining (MMEJ) leading to targeted mutagenesis is approximately two orders of magnitude more frequent than repair by homologous recombination (HR) using a repair matrix (Huang and Puchta, 2019). More recently, new variants of the CRISPR/Cas9 technology such as base editing or prime editing have emerged that allow to predetermine the precise nature of the mutation, albeit with certain limitations. These variants are based on a nickase version of the Cas9 that cuts only one and not both DNA strands, and which is fused to a protein domain with enzymatic action, for example to a cytidine and/or adenine deaminase domain for C and/or A base editing (Shimatani et al., 2017; Zong et al., 2017; Yan et al., 2018; Li et al., 2020), or to a reverse transcriptase domain for prime editing (Hua et al., 2020; Lin et al., 2020; Xu et al., 2022).

Another limitation of the initial CRISPR/Cas9 technology was the protospacer adjacent motif (PAM), *i.e.* the need for the triplet NGG downstream of the targeted site in the genome. Both the use of other RNA-guided nuclease such as Cas12a/CPF1 with its PAM sequence TTTN located upstream of the target (Zetsche et al., 2015), and the molecular engineering of Cas9 leading to the xCas9 (Hu et al., 2018) and Cas9-NG variants (Nishimasu et al., 2018) markedly enlarged the number of sites amenable to genome editing in a given genome. After initial exemplification in human cell lines, all of these improvements have been successfully transferred to plants and are now available for plant genome editing (Chen et al., 2019), including the latest development referred to as PAM-less genome editing (Ren et al., 2021).

The production of edited plants is a time consuming and labor-intensive process, which generally involves the *in vitro* culture of hundreds of calli over several months. This created a need for rapid, reliable and cost-efficient evaluation methods both for the implementation of novel genome editing tools and the day to day test of sgRNA designs. In fact, in maize, for example, CRISPR/Cas9-mediated mutation rates show important variations between genes and between guides in a given gene (Doll et al., 2019), despite ever improving bioinformatics tools for the design of sgRNAs. With a size of 2.3 Gb and over 32,000 predicted genes the B73 maize reference genome is of intermediate size for angiosperms and behaves as a diploid despite important remnants of its allotetraploid origin (Schnable et al., 2009). Protoplasts are an attractive test system, since a large number of cells can be transformed in parallel to provide in depth insight in the efficiency of a molecular construct within one or two days (Lin et al., 2018). In maize, protoplast systems have been used for the initial setup of the technology with a marker gene (Feng et al., 2016), the codon-optimization of the Cas9 protein and the validation of an endogenous maize U6 snRNA promoter (Zhu et al., 2016), the test of new vector sets (Xing et al., 2014; Gentzel et al., 2020), the establishment of a DNA-free protocol based on pre-assembled ribonucleoprotein complexes (RNPs) composed of purified recombinant Cas9 enzyme and *in vitro* transcribed guide RNA (gRNA) molecules (Sant'Ana et al., 2020) and the evaluation of targeted base editing (Zong et al., 2017).

Here we used a streamlined maize protoplast system coupled to a novel NGS analysis pipeline to evaluate the efficiency of different sgRNA scaffolds, novel Cas9 variants with relaxed PAM sequences and cytidine base editing. Selected constructs were also used in stable maize transformation.

## Materials and methods

### Plant material and growth conditions

The maize (*Zea mays*) inbred line A188 (Gerdes and Tracy, 1993) and derived transgenic or edited plants were grown in 15 m^2^ growth chambers that fulfil the French S2 safety standards for the culture of transgenic plants (Gilles et al., 2021). The photoperiod consisted of 16 h light and 8 h darkness in a 24 h diurnal cycle. Temperature was set to 26°C/17°C (day/night) during the first 3 months after sowing and then to 28°C/19°C for the remaining month of the life cycle. The relative humidity was controlled at 55% (day) and 65% (night). Seeds were germinated in 0.2 l of Favorit MP Godets substrate (Eriterre, Saint-André-de-Corcy) and transferred after 2 weeks to 8 l of Favorit Argile TM + 20% perlite substrate (Eriterre, Saint-André-de-Corcy) and watered with a nutritional solution composed of 1.2 g/l Peters^®^ Excel Hard Water Grow Special 18-10-1+2 MgO+TE (ICL Limas, France) and 0.04 g/l Micromax (ICL Limas, France). The insertional mutant *Zmicea::Mu* (UFmu-02855) of the UniformMU collection (Settles et al., 2007) was obtained from the stock center of the maize genetics cooperation. All plants were propagated by hand pollination.

### Protoplast extraction and transformation

**Figure 1:**
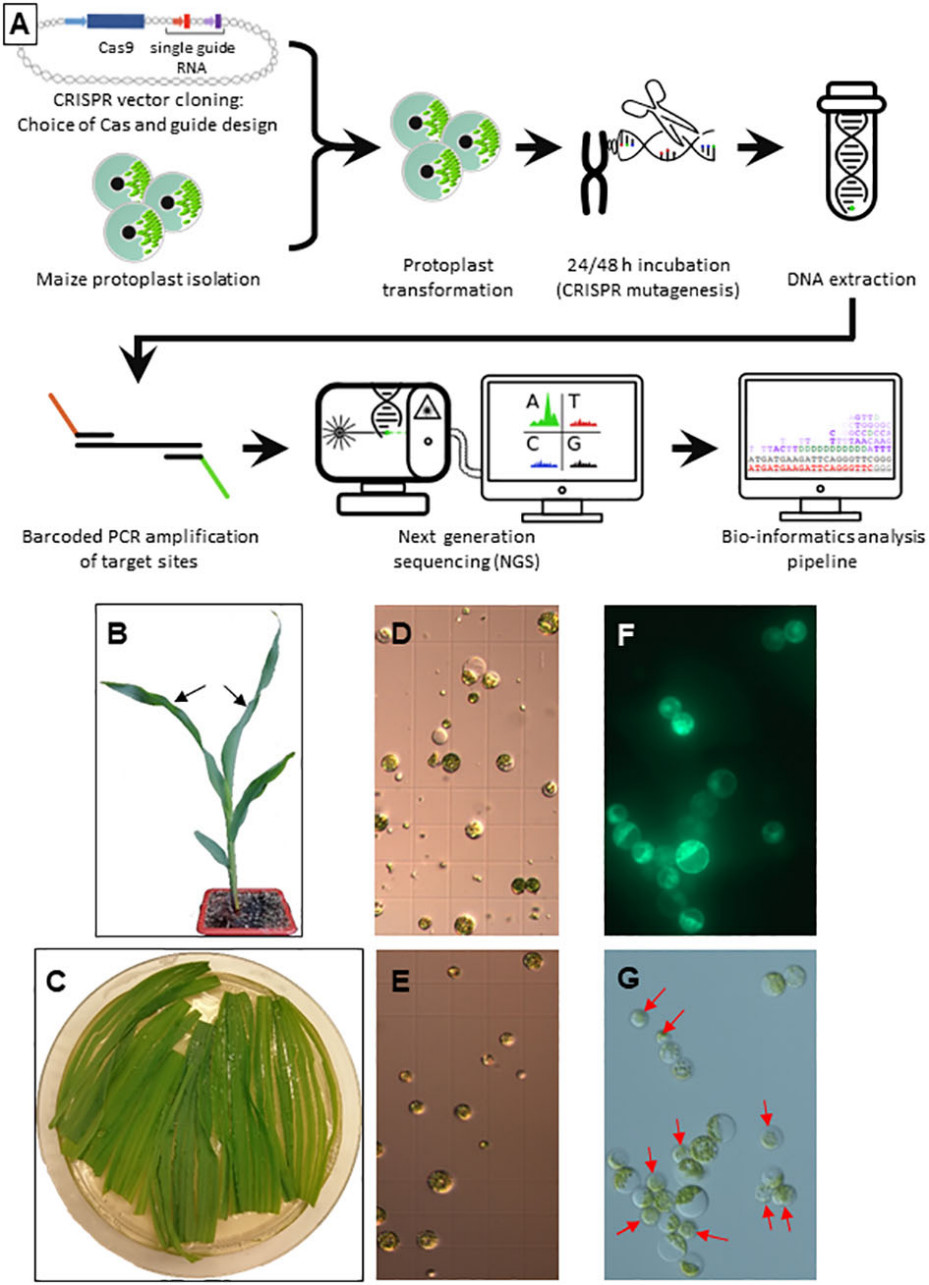
Protoplast isolation and transformation **(A)** Overview of the developed workflow. **(B)** Germination at 4 leaf stage. The two youngest leaves used for protoplast isolation are indicated by black arrows. **(C)** Longitudinally sliced leaf pieces during enzymatic digestion. **(D, E)** Protoplast preparations before **(D)** and after **(E)** purification on a sucrose gradient. **(F, G)** Protoplasts 48 h after transformation with a GFP control plasmid in UV light **(F)** and visible light **(G)**. Red arrows indicate non-transformed protoplasts.

Maize protoplast extraction and transformation were performed with a protocol adapted from (Wolter et al., 2017) with line A188 (**Figure 1A**). Briefly, 12-15 days old seedlings were grown in soil with a 16 h photoperiod and the youngest fully expanded leaves (**Figure 1B**) of 4 healthy plants were transferred into a 94 mm Petri dish with 15 ml of enzyme mix (0.6 mannitol, 10mM MES pH 5.7, 1.5% w/v Cellulase R-10, 0.75% w/v Macerozyme R-10, 0.1% w/v Pectolyase Y-23, 10mM CaCl_2_, 0.1% v/v BSA) and cut in 1 mm stripes parallel to the midrib (**Figure 1C**). The stripes were arranged in a monolayer and vacuum infiltrated at -500 mbar for 30 min in the dark at room temperature followed by incubation at 26°C with shaking (20 rpm) for 4 h.

The protoplasts were filtered through a 70 µm cell strainer, collected by centrifugation at 100 g and resuspended in 2 ml W5 buffer (154 mM NaCl, 125 mM CaCl_2_, 5 mM KCl, 2 mM MES pH 5.7, **Figure 1D**). The protoplasts were layered on a sucrose cushion (Banks and Evans, 1976) and centrifuged for 10 min at 200 g to eliminate cell debris (**Figure 1E**). The protoplasts were washed in four times their volume of W5, centrifuged for 5 min at 100 g, resuspended in W5 and put on ice for 30 min. In the meantime, the protoplasts were counted, usually yielding 3-4 x 10^6^ cells. The protoplasts were centrifuged at 100 g for 5 min and resuspended in MMG buffer (0.4 M mannitol, 15 mM MgCl_2_, 4 mM MES pH5.7) to a 2.5 x 10^6^ cells ml^-1^ density.

Each transformation was performed in a 2 ml Eppendorf tube adding the three following solutions in that order, mixing gently but thoroughly: 500 000 protoplasts (200 µl), 1.62 x 10^23^ copies of plasmid DNA (NucleoBond^®^ Xtra Maxi, Machery-Nagel Hœrdt, France) and 250 µl polyethylene glycol (PEG) solution (40% w/v PEG 4000, 0.2 M mannitol, 0.1 M CaCl_2_). After 15 min of incubation in the dark at room temperature, 800 µl of W5 was added and the tubes were centrifuged for 3 min at 100 g. The pellet was resuspended in 2 ml W5 and the protoplasts were transferred in 24 well cell culture plates and incubated for 48 h at 26°C in the dark.

The protoplast transformation efficiency was calculated by dividing the number of cells expressing green fluorescent protein (GFP; parallel transformation with plasmid L1036 promoting GFP expression under the control of the constitutive cassava vein mosaic virus (*CsVMV*) promoter, **Figure 1F**) by the total number of viable cells using an Axio Imager M2 fluorescence microscope (Zeiss, **Figure 1G**). Protoplasts were pelleted for 3 min at 100 g and the pellets stored at -80°C.

### Vectors for targeted mutagenesis and base editing

The original vectors harboring different Cas9 derivatives and/or scaffolds (**Supplementary Table S1**) were derived from L1609, an integrative plasmid harboring the Cas9 cassette, an empty site for sgRNA1 and a Basta resistance cassette, and L1611, a small plasmid used for initial cloning of sgRNA2 (Doll et al., 2019).

### Stable maize transformation

Agrobacterium-mediated transformation of inbred line A188 was performed according to a published protocol (Ishida et al., 2007). Briefly, immature 13 DAP embryos were co-cultivated with Agrobacterium and glufosinate-resistant type I calli (hard and compact) selected on auxin containing media. After suppression of auxin, shoots were initiated in the presence of cytokinin and gibberellin inhibitors. Roots were obtained in the absence of hormones. Finally, the plantlets were transferred to soil (see above). The precise composition of the different *in vitro* culture media and the respective incubation times are summarized in **Supplementary Table S2**.

### DNA extraction and amplification

DNA extractions from transformed protoplasts (500 000 protoplasts) or from leaf punches (5 punches of 25 mm^2^) of stably transformed plantlets (10 DAS) were performed with a Biosprint 96 robot (Qiagen) and a DNeasy 96 plant kit (Qiagen). The gene-specific parts of the primers (**Supplementary Figure S1**) and melting temperatures used to amplify the target regions around the CRISPR/Cas9 binding site with Phusion™ High-Fidelity DNA Polymerase (Thermo Scientific) are indicated in **Supplementary Table S3**.

### Molecular characterization of stable maize transformants

Transfer DNA (T-DNA) integrity was checked as previously described (Gilles et al., 2017). Molecular characterization of the sites targeted by genome editing involved, for each targeted gene, PCR amplification with specific primers (**Supplementary Table S3**) on DNA extracted from leaves of T0 plants, followed by Sanger sequencing. In T1 plants segregation of Cas9 bearing T-DNA was evaluated by PCR amplification of the *Bar* gene, checking the presence and quality of genomic DNA by PCR amplification of the GRMZM2G136559 (Zm00001eb386680) control gene (Doll et al., 2019).

### Library construction and sequencing

Protoplast PCR products spanning the CRISPR/Cas9 target site and carrying tails with homology to NGS adapters (**Supplementary Figure S1**) were gel purified, cleaned with the NucleoSpin Gel and PCR Clean-up kit (Machery-Nagel Hœrdt, France) and quantified with the Qubit dsDNA high-sensitivity assay (Thermo-Fischer). Libraries were constructed with Index5/Index7 adapters, quality controlled with a High Sensitivity D1000 ScreenTape Assay on an Agilent Tapestation and sequenced in multiplex (12 libraries) with a NextSeq 500/550 Mid Output v2 kit (300 cycles) on an Illumina NextSeq500 platform in paired-end mode.

### NGS analysis

**Figure 2:**
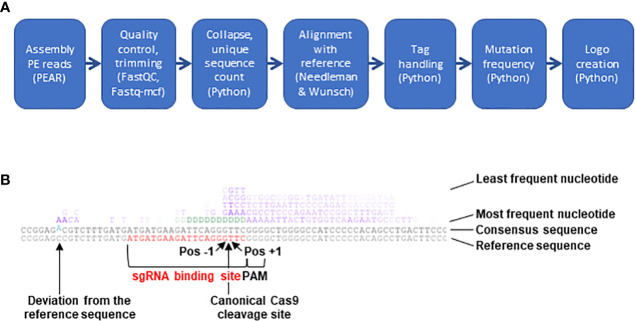
NGS analysis pipeline. **(A)** Schematic presentation of the 7 steps constituting the bioinformatics pipeline built to analyze CRISPR/Cas9-mediated mutations. **(B)** Example of mutation logo. The logo shows from the bottom to the top the reference sequence (sgRNA binding site in red), the consensus of all sequences (differences to the reference sequence in blue and offset) and the most frequent nudeotide at mutated sites. The following lines represent other nucleotides with decreasing frequency. In the upper 4 lines insertions and mismatches are indicated by the four bases G, A, T and C (in purple) and deletions (absence of a base at a given position) by aD {in green). The intensity of each letter is proportional to its frequency. The position of the canonical Cas9 cleavage site 3 nudeotides upstream of the PAM is indicated by an arrow. Pos-1, first base to the left of the cleavage site; Pos+1, first base to the right of the cleavage site.

Software of the Illumina NextSeq500 platform was used to assign raw read sequences to libraries based on the indexes and to trim the NGS adapters. For subsequent analysis, a 7-step bioinformatics pipeline mixing existing programs and custom-made scripts was built (**Figure 2**, https://gitbio.ens-lyon.fr/rdp/crispr_proto_maize). In the first step the paired-end reads were assembled with PEAR software (Zhang et al., 2014, version 0.9.10). The assembled reads were quality checked using fastQC (http://www.bioinformatics.babraham.ac.uk/projects/fastqc, version 0.11.7) and trimmed using fastq-mcf (https://github.com/ExpressionAnalysis/ea-utils, version 1.04.676). To avoid problems in downstream sequence alignments, sequences containing one or several undetermined nucleotides (N) after this step (on average, 0.02% of the output sequences) were eliminated from further analysis despite their overall acceptable quality. For the third step of the pipeline a specifically developed Python program was used to trim the 5-nt tags at both extremities of the sequence and to concatenate their sequences to the sequence name. This program then identified identical sequences, counted their number of occurrences and adjusted this count if not only the sequences but also the tags were identical, *i.e.* the sequences were PCR duplicates, corresponding in fact to a single initial editing event. At the end of this step the obtained file (in FASTA format) contained one sequence for each group of identical sequences and their counts. The next step consisted in the pairwise alignment of the representatives of the different groups of reads with the reference sequence using the Needleman & Wunsch algorithm, as implemented in the 'needle' program from EMBOSS (Needleman and Wunsch, 1970, Rice et al., 2000; version 6.6.0.0, with scoring matrix EDNAFULL83 and options “-gapopen 10.0 -gapextend 0.5”). After the alignment, another Python program identified the different mutations and computed their frequencies. These quantitative data contained in this file were used in the last step of the pipeline to create a logo (**Figure 2**).

## Results

### Coupling of protoplast transformation with NGS provides deep insight in the CRISPR/Cas9 mutation landscape

To reliably evaluate genome editing by CRISPR/Cas9 in maize protoplasts, a robust experimental system yielding protoplasts with a good viability at high density was established. Comparative tests of several parameters incited us to use as starting material young leaves of soil born germinations, which were easier to obtain in large quantity and without the risk of contamination than *in vitro* germinations or Black Mexican Sweet (BMS) cell cultures (**Figure 1**). Other important choices to increase the overall yield and/or viability were the tenderness of the leaves (the two youngest leaves at the 4 to 5 leaf stage, **Figure 1B**), the addition of pectolyase to the enzyme mix containing also cellulase and macerozyme, the vacuum infiltration of the enzyme mix, a purification step on a sucrose cushion, the use of polyethylene glycol (PEG) rather than electroporation for DNA uptake, ultrapure plasmid DNA without salts and simple deep freezing rather than grinding of protoplasts prior to DNA extraction (see Materials and Methods for details). The transformation rate was calculated by transforming a protoplast aliquot with a plasmid expressing a *GFP* reporter gene under the control of the constitutive *CsVMV* promoter (**Figure 1**).

After incubation for 24 to 48 h allowing transcription, translation and action of the CRISPR/Cas9 tool, total DNA was extracted from protoplasts. To assess the different types of mutations caused by a given construct in a pool of protoplasts, the target site was amplified with a proof-reading enzyme by site-specific PCR and the PCR product subjected to NGS sequencing (**Figure 1A**). In addition to the maize-specific part, the primers contained a 5 nt tag with a random sequence and part of the Illumina adapter (**Supplementary Figure S1**). The random nature of the tag allowed to distinguish NGS sequence reads originating from independent amplifications of the target site (different tags) or representing the same PCR product (identical tag). This tag is not to be confused with the index added during the second amplification with the full Illumina adapter, which allowed multiplexing of libraries in a single flow cell. The raw sequence data obtained were deposited at EBI under the accession number PRJEB56234.

To analyze the type and frequency of CRISPR/Cas9-mediated mutations, a 7-step bioinformatics pipeline combining existing programs and custom-made scripts was developed (**Figure 2A**) and made available (https://gitbio.ens-lyon.fr/rdp/crispr_proto_maize). The overlapping paired-end mode was chosen to enhance sequence quality and allow the processing of slightly larger PCR products compared to single read mode. After classical pairing with PEAR (Zhang et al., 2014), quality control with FASTQC (Andrews, 2010) and trimming of remaining adapter sequences with Fastq-mcf (Aronesty, 2013), the tags at the 5’ and 3’ ends of the sequences were removed with a Python script and added to the sequence name. In the next step, unique sequences were counted and extracted for alignment with the non-mutated reference sequence. After testing several alternatives such as BLAST, Bowtie or Smith & Waterman, pairwise alignments with the reference sequence were performed with the Needleman & Wunsch algorithm using a custom score matrix and suitable gap opening and extension penalties, since it (i) allowed systematic alignment over the entire length of the reference sequence and (ii) satisfactorily handled even important size differences between the mutated and the reference sequence. The next step allowed to quantify by a Python script the different types of mutations (deletion, insertion, mismatch) for each position of the reference sequence, excluding the primer regions and distinguishing the 20 nt CRISPR/Cas9 range corresponding to the sgRNA binding site from the rest of the amplified sequence. Finally, the types and positions of the mutations were summarized in a logo (**Figure 2B**). Together with an original assembly of the tools, the logo was the most distinctive feature of the pipeline (**Supplementary Table S4**, Guell et al., 2014; Boel et al., 2016; Park et al., 2016; Pinello et al., 2016; Wang et al., 2017; Liu et al., 2019).

This experimental system was used to analyze the CRISPR/Cas9 mutation landscape at 14 different target sites (**Supplementary Table S3**). On average, nearly 15 million sequence reads were obtained for each target. An average 99.2% success rate of the PEAR step and a 99.3% success rate of the combined FASTQC/Fastq-mcf step were indicators for excellent sequence quality (**Supplementary Table S5**). The collapse to unique sequences reduced the number of reads to 11% on average, rendering the time-consuming pairwise alignment step easily feasible. After several tests, the (modifiable) default minimal value of the Needleman & Wunsch score needed for a sequence to be retained for subsequent steps was fixed to 200, which was a compromise between exhaustiveness to include even large deletions or insertions and specificity to exclude PCR products not related to the target site. When applying this default threshold, on average 8.2% of the unique sequences were eliminated (**Supplementary Table S5**). The next step was to count the occurrences for each unique sequence in the initial read sets either with or without consideration of the 5-nt tag. Considering the mutation rate at every single position of the 14 analyzed amplicons, the highest difference ever observed with or without consideration of the tag was a 2.1-fold decrease when considering the 5 nt-tag. This suggested that there was no strong over-representation of particular PCR products and that the relative values obtained with or without tag were very similar. In the last step the table with numerical values was exploited to create a visual representation of the results (mutation logo, as exemplified in **Figure 2B**). With regard to the canonical Cas9 cleavage site 3 nucleotides upstream of the PAM site, the positions of mutations will be indicated with increasing negative or positive numbers to the left (upstream) or to the right (downstream) of the cleavage site throughout this manuscript (**Figure 2B**).

Theoretical considerations indicate that the observed final counts are only semi-quantitative values under our experimental setup. The transformation of 500 000 protoplasts allows at the most 1 million (diploid genome) independent mutations. Since each amplicon was sequenced with a depth of 15 million reads, this indicates that on average a given mutation was independently amplified 15 times with different 5-nt tags. Considering that only 80 ng of protoplast DNA (containing approximately 66 000 genomes) was amplified, the real effect was even much stronger. This limitation needs to be kept in mind when analyzing the numbers presented in the following chapters.

### Mutagenesis tendencies in selected *ZmSWEET* genes

In order to validate our quick transient protoplast transformation to gather information on CRISPR/Cas9 efficiency, we targeted three genes from the *Sugars Will Eventually Be Exported Transporter* (*SWEET*) family, previously identified as expressed at an embryo/endosperm interface (Doll et al., 2020): *ZmSWEET14a* (Zm00001e011125), *ZmSWEET14b* (Zm00001e021494) and *ZmSWEET15a* (Zm00001e022582). Each gene was targeted with two sgRNAs, which were identical for the paralogous genes *ZmSWEET14a* and *ZmSWEET14b* showing very high sequence homology (**Supplementary Table S3**). For NGS analysis, gene specific primers were designed to amplify the two targets in *ZmSWEET14a* and in *ZmSWEET14b* with a single amplicon of 199 bp and 194 bp, respectively. For *ZmSWEET15a*, only the mutagenesis events at the sgRNA1 target were analyzed.

To assess the general sgRNA design efficiency, the occurrence of mutations (deletions, insertions and mismatches) starting or ending within the 20-nt target regions was compared to the occurrence of mutations originating outside of the targets. All five targets considered, the number of deletions and insertions per base was at least 11 times and up to 567 times higher within the 20-nt target than in the rest of the amplicon. The number of mismatches was also slightly higher within all targets except for the sgRNA2 target in *ZmSWEET14a* (**Supplementary Table S5**). Please note that sgRNA1 contained an additional A at its 5'-end, which was not present at the genomic target site, to allow efficient transcription from the OsU3 promoter (**Supplementary Table S3**).

**Figure 3:**
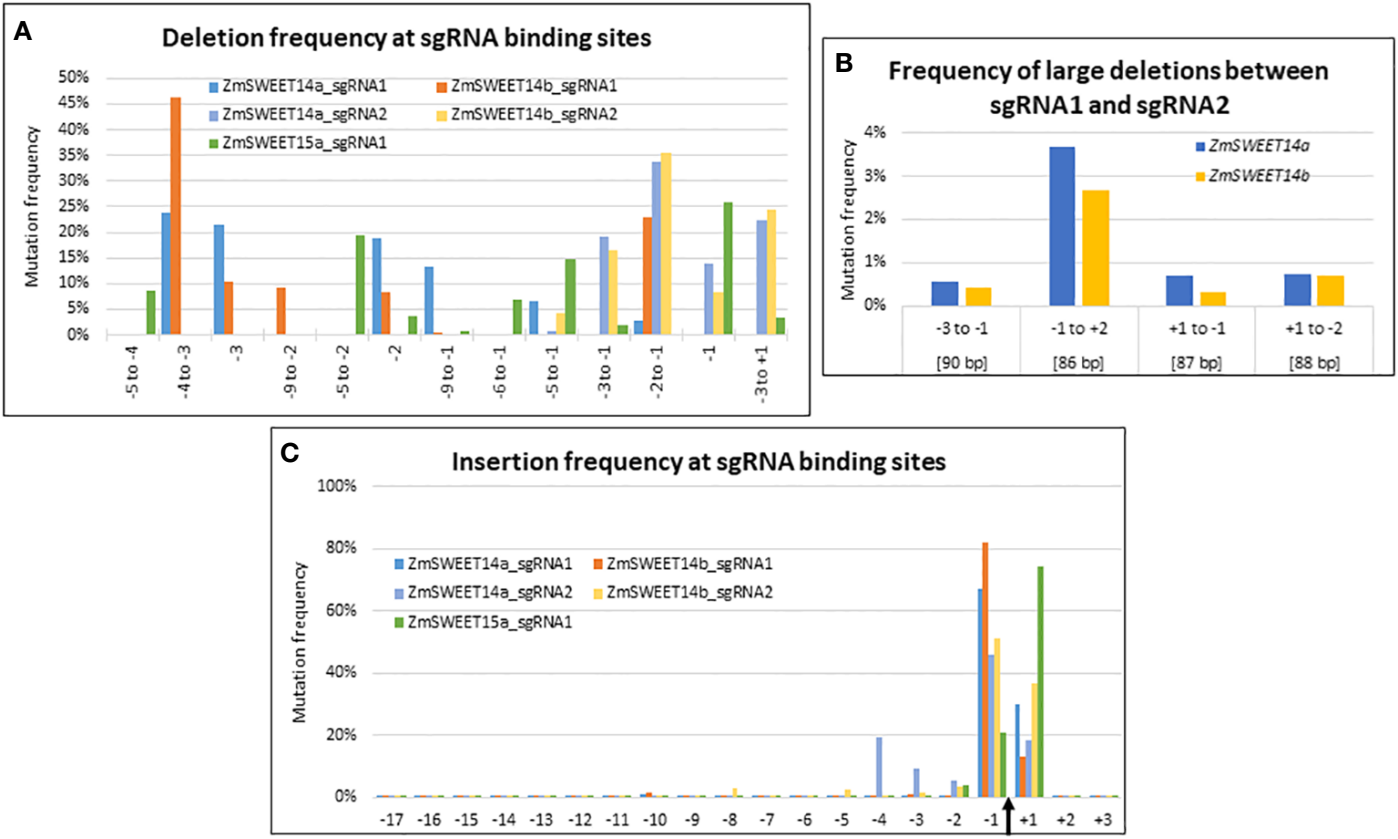
CRISPR/Cas9-induced mutations landscape in ZmSWEET genes. **(A–C)** Graphs indicating the deletion and insertion frequency in Zm00001e011125 (ZmSWEET14a), Zm00001e021494 (ZmSWEET14b) and Zm00001e022582 (ZmSWEET15a). **(A)** Frequency of selected deletions. **(B)** Frequencies of large deletions observed between the two target sites in Zm00001e011125 (ZmSWEET14a) and Zm00001e021494 (ZmSWEET14b). Positions are relative to the Cas9 cleavage sites of each target. Values in brackets indicate the length of the deletions. **(C)** Frequency of insertions at all positions of the target sequences. The Cas9 cleavage site is indicated by a black arrow. The numbers of the x-axis indicate the position of the mutations relative to this cleavage site.

Although the sgRNA target sequences were identical between *ZmSWEET14a* and *ZmSWEET14b* the type of deletions observed within the target range of sgRNA1 showed contrasted results between the two genes (**Figure 3A**). For example, a deletion at position -2 to -1 was 10 times more frequent for *ZmSWEET14b* as compared to *ZmSWEET14a*. In contrast, the type of deletions observed at sgRNA2 target followed the same trends for the two genes with the highest frequency observed for a CC deletion at position -2 to -1 (**Figure 3A**). For *ZmSWEET15a*, the usual -1 position had the highest deletion frequency (25%), followed by positions -5 to -2 (20%) and -5 to -1 (15%) (**Figure 3A**). The efficiency of sgRNA2 (~85%) to generate deletions in *ZmSWEET14a* and *ZmSWEET14b* was far greater than the one of sgRNA1 (~9%) (**Supplementary Figure S2A**).

Large deletions between the two targets sites of *ZmSWEET14a* and *ZmSWEET14b* represented ~5% of all deletions observed for each gene, with the highest frequency observed for the 86 bp deletion between position -1 of the first target and position +2 of the second target (**Figure 3B**).

The vast majority of insertions concerned positions -1 and +1 for all 5 targets, the values at position -1 being higher with the exception of *ZmSWEET15a* (**Figure 3C**). There was a good correspondence for sgRNA1 and sgRNA2 targets between *ZmSWEET14a* and *ZmSWEET14b*. The efficiencies of the two sgRNAs to generate insertions in *ZmSWEET14a* and *ZmSWEET14b* were balanced in *ZmSWEET14a* with ~47% and ~53%, whereas sgRNA2 was more efficient in *ZmSWEET14b* (~77%) than in *ZmSWEET14a* (~23%). The imbalance for sgRNA2 was reminiscent of the one observed for deletions (**Supplementary Figure S2B**).

The two vectors used to target *ZmSWEET14a*, *ZmSWEET14b* and *ZmSWEET15a* in protoplasts were used in parallel to generate stable transformants (**Supplementary Table S6**). All nine T0 *Zmsweet14a*/*14b* mutant plants bared mutations at the sgRNA2 target site for each gene and only one plant had a mutation at the sgRNA1 target site in *ZmSWEET14b* (a 1 bp insertion at position -1). The analysis of 8 events (for one event Sanger sequencing could not be interpreted) at the sgRNA2 target site in *ZmSWEET14a* revealed that all events were bi-allelic. The most frequent ones with 62.5% (10/16) and 18.75% (3/16) were a 1 bp insertion at position -1 and a 1 bp deletion at position -1, respectively. In *ZmSWEET14b*, the most frequent types of mutation were a 1 bp insertion at position -1 (20%), a 5 bp deletion at position -2 to +3 (20%) and a 90 bp deletion (20%) not located between the two targets. In the case of *ZmSWEET15a* only one plant was retrieved after stable genetic transformation, and this plant carried a 23 bp deletion at the sgRNA1 target site (**Supplementary Table S6**). These numbers obtained in stable maize transformation of immature embryos are not statistically significant due to small sample size but fit the overall trends observed in the leaf protoplast system.

### Similar efficiency of three different sgRNA scaffolds

**Figure 4:**
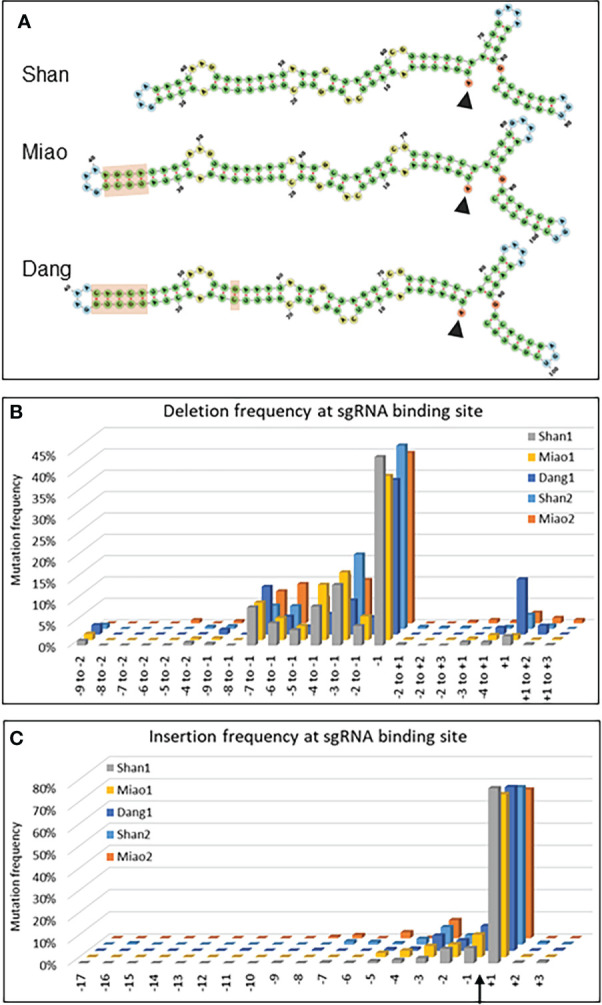
Evaluation of sgRNA scaffolds. **(A)** Secondary structures of the sgRNAs composed of the 20 nucleotides complementary to the target sequence in Zm00001e008508 (start indicated by arrowhead) and the three different scaffolds indicated. The light brown rectangles highlight the differences between the scaffolds. **(B, C)** Graphs indicating the deletion **(B)** and insertion **(C)** frequency of mutations for selected deletions **(B)** and insertions at all positions **(C)** of the 20 nt target sequence of Zm00001e008508. The Cas9 cleavage site is indicated by an arrow. Shan1 and Shan2, as well as Miao1 and Miao2, are biological replicates, i.e. represent data from two independent experiments carried out at several months’ interval with different protoplast batches and independent DNA extraction, amplification and NGS analysis.

One of the major changes in adapting the naturally occurring type II CRISPR/Cas9 bacterial defense system to a biotechnology tool was the fusion of the crRNA and a normally trans-encoded tracrRNA into a single sgRNA molecule capable to form a complex with the Cas9 protein and to sequence-specifically cleave target DNA (Jinek et al., 2012; Cong et al., 2013). Several designs of sgRNA scaffolds have been proposed mainly differing in the length and structure of the hairpin linking the two initial molecules (**Supplementary Figure S3**). To compare the most frequently used scaffold in plants (Shan et al., 2013) with two alternative designs (Miao et al., 2013; Dang et al., 2015), the same 20 nucleotides complementary to the target sequence in Zm00001e008508 was linked to the three different scaffolds called hereafter Shan, Miao and Dang (**Figure 4A** and **Supplementary Table S3**). All three scaffolds were transformed in parallel into aliquots of a single batch of maize protoplasts (data set 1: Shan1, Miao1 and Dang1) and the experiment was repeated for the Shan and Miao scaffolds several months later (data set 2: Shan2 and Miao2).

In a first instance, the type of scaffold may influence the rate limiting step of double stranded breaks which is the formation of a ternary complex between Cas9/sgRNA and DNA (Raper et al., 2018). Using the ratio of mutations within over outside the target range as an indicator of CRISPR/Cas9 efficiency, all three scaffolds resulted in efficient targeted mutagenesis (**Supplementary Table S5**, **Supplementary Figure S3**). In the first experiment (single protoplast batch for three constructs), the Dang scaffold showed a lower deletion efficiency (31-fold increase within the target range) than the Shan (99-fold) and Miao scaffolds (128-fold). For insertions, the efficiency was quite similar between the Dang (42-fold), Shan (31-fold) and Miao scaffolds (35-fold) There were no tangible differences between scaffolds for mismatch mutations (0.35, 0.37 and 0.37-fold increase respectively, **Supplementary Table S5**).

In addition, the nature of the Cas9/sgRNA complex can have an influence on the type of double strand break (blunt versus staggered), which in turn influences the repair mechanisms involved and finally the type of mutations (Molla and Yang, 2020). A closer look at the type and position of the deletions and insertions revealed a remarkable difference of the Dang scaffold for single base deletion at the +1 position (13%) compared to the Shan (2%) and Miao (1%) scaffolds (**Figure 4B**). The frequency remained nevertheless lower than at the usual -1 position (36%). Despite some minor quantitative differences, the overall pattern for other deletions as well as for insertions was quite similar between repetitions and between scaffolds (**Figures 4B, C**).

Finally, the fully independent repetition of the experiment for the Shan and Miao scaffolds showed that the mutation rates within and outside the target range were more different between experiments than between scaffolds (**Supplementary Table S5**) suggesting that (semi)-quantitative comparisons need to be carried out in a single experiment with parallel plasmid DNA isolation, the same batch of protoplasts, parallel NGS library construction and the same Illumina flow cell.

### Cas9 variants with relaxed PAM sites work in maize

**Figure 5:**
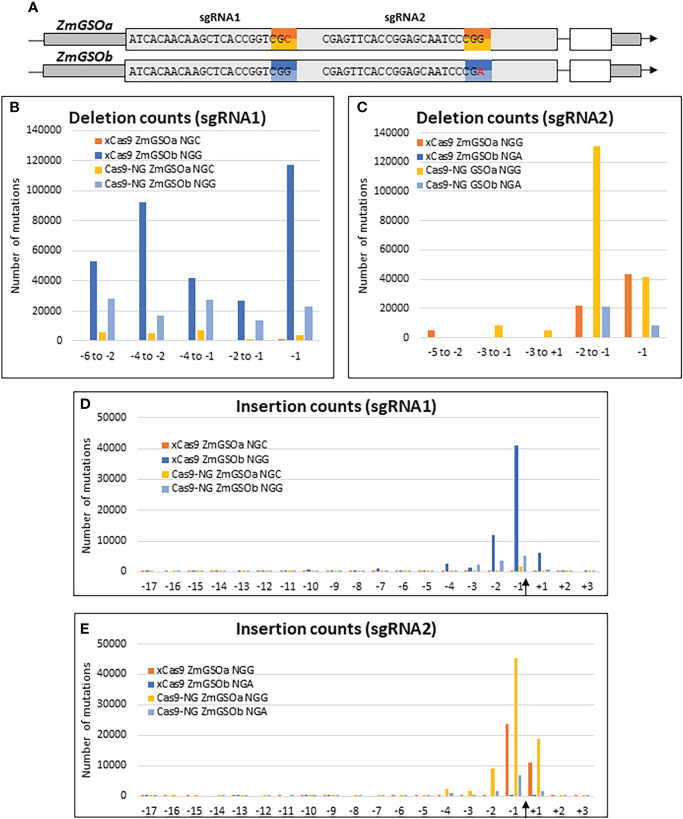
Efficiency of Cas9 variants. **(A)** Gene models of *ZmGSOa* and *ZmGSOb* indicating the target sequences and PAM sites for sgRNA1 and sgRNA2. **(B-E)** Graphs indicating the number of mutations for selected deletions **(B, C)** and insertions at all positions of the 20 nt targets **(D, E)** at the binding sites of sgRNA1 and sgRNA2 in *ZmGSOa* (NGC and NGG PAM) and *ZmGSOb* (NGG and NGA PAM) generated by xCas9 and Cas9-NG. The Cas9 cleavage site is indicated by an arrow.

The recent development of engineered Cas9 proteins with relaxed PAM sequences has alleviated the limitation of the strict NGG PAM sequence of the original SpCas9 system and given access to larger portions of genomes for genome editing. To assess the relative efficiencies of the xCas9 (Hu et al., 2018) and Cas9-NG (Nishimasu et al., 2018) variants, we took advantage of the high sequence similarity between the two paralog genes *ZmGASSHOa* (*ZmGSOa*, Zm00001e035023) and *ZmGASSHOb* (*ZmGSOb*, Zm00001e010407) to design two sgRNAs, each of them targeting the same string of 20 nt in both *ZmGSO* genes followed by either the canonical NGG PAM in one of the *ZmGSO* genes or an alternative NG PAM (CGC or CGA) in the other *ZmGSO* gene (**Figure 5A**). Protoplast transformation was performed with individual constructs for each Cas9 alternative and resulted in typical small insertions and deletions around the Cas9 cleavage site for both systems (**Figure 5** and **Supplementary Figure S4**).

Both xCas9 and Cas9-NG actually provoked deletions at the non-canonical NGC and NGA PAM sites. However, the number of deletions for 5 selected intervals (**Figures 5B, C**) was on average 5 times (Cas9-NG) and 101 times (xCas9) lower than for the canonical NGG PAM at the sgRNA1 target site and on average 10 times (Cas9-NG) and 2980 times (xCas9) lower at the sgRNA2 target site. Similarly, the number of insertions at positions -3, -2, -1 and +1 (**Figures 5D, E**) was on average 35 times (Cas9-NG) and 87 times (xCas9) lower than for the canonical NGG PAM at the sgRNA1 target site and on average 11 times (Cas9-NG) and 2017 times (xCas9) lower at the sgRNA2 target site. Taken together these results indicate that the capacity to induce indels at NG PAM sites as compared to NGG PAM sites is one order of magnitude lower for Cas9-NG and two to three orders of magnitude lower for xCas9.

The relative frequencies of selected deletions were very similar for the NGG/NGC context for both xCas9 (at the most 1.68-fold) and Cas9-NG (at the most 1.91-fold, **Supplementary Figure S4A**), whereas more substantial differences existed for the NGG/NGA context for xCas9 (maximum 41-fold at positions –5 to -2) and Cas9-NG (maximum 25-fold at positions -2 to -1) **Supplementary Figure S4B**). Insertions were most frequent at the -1 position followed by the -2 and either the -3 or +1 position for the NGG/NGC context and by the +1 position for the NGG/NGA context. Differences affecting only one Cas9 variant in a given NGG/NG context may reflect differences in Cas9 positioning at the target site due to sub-optimal engineering.

### APOBEC1 C-deaminase permits efficient base editing in maize

Base editing has emerged as an efficient albeit more limited alternative to gene editing by homologous recombination (Mishra et al., 2020). For C to T editing, two main sources for cytidine deaminases have been successfully used in plants, APOBEC1 from rat (Zong et al., 2017) and CDA1 from sea lamprey (Shimatani et al., 2017). In this study the nCas9-PBE (plant base editor) was used, in which the APOBEC1 domain is fused to a Cas9-D10A nickase and an uracil glycosylase inhibitor (UGI). The presence of UGI avoids an abasic site and error-prone repair and favors mismatch repair of the nicked strand (Komor et al., 2016). The target sites were chosen in Zm00001e018755 (*ZmICEa*) and Zm00001e008118 (*ZmZOU/O11*), two transcription factors of the bHLH family (Grimault et al., 2015; Feng and Song, 2018). The goals were to create a STOP codon (Q274/, target 1) and to mutate the only serine predicted *in silico* to be phosphorylated (Walley et al., 2016)(S283L, target 2) in ZmICEa, as well as to create a STOP codon (Q355/) in ZmZOU.

**Figure 6:**
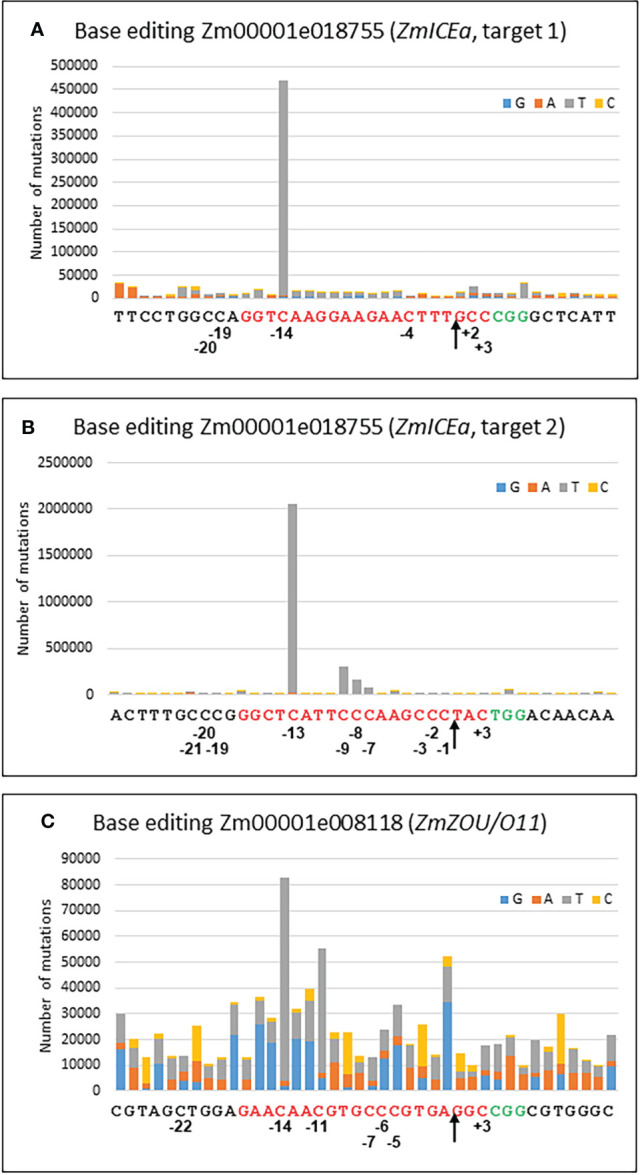
Base editing. Cumulative graph indicating the number and type of mutations for every position of the 20 nt target sequence (red) and the 10 nt upstream and downstream for target 1 **(A)** and target 2 **(B)** of Zm00001e018755 and for Zm00001e008118 **(C)**. The PAM site is in green. Positions of selected bases refer to the nCas9 nick site (arrow).

Protoplast transformation with individual constructs for each of the three targets (**Supplementary Table S3**) resulted in efficient base editing for the two targets in Zm00001e018755 (*ZmICEa*) and moderate base editing for Zm00001e008118 (*ZmZOU/O11*, **Figure 6**). C to T transitions were by far the most frequently observed mismatches in the sgRNA binding site of 20 nt with 84% for target 1 of *ZmICEa*, 94% for target 2 of *ZmICEa* and 47% for *ZmZOU/O11* (**Supplementary Figure S5**). The frequency of other nCas9 induced mismatches was very low, since the considerable background level of mismatches likely caused by errors introduced during PCR and Illumina NGS reactions (Schirmer et al., 2015) was quite similar within and outside of the sgRNA binding range, with a ratio of 0.62, 1.04 and 1.28 for the three targets (**Supplementary Figure S5**). Deletions and insertion were respectively one and two orders of magnitude less frequent than mismatches and there was no notable difference between their frequency in the sgRNA binding range and neighboring regions, indicating that the D10A mutation of the Cas9 efficiently reduced or aborted Cas9-mediated double strand break and subsequent NHEJ. Deletions were most frequent in homopolymer stretches, likely reflecting Illumina NGS errors.

The frequency of C to T transitions strongly varied with distance from the nCas9 nick site. For example, in the case of Zm00001e018755 (*ZmICEa*, target 2) C to T base editing gradually increased for positions -7, -8, -9 and -13, whereas no substantial base editing occurred at positions -1, -2, -3 and -15 (**Figure 6**). Similarly, editing was highest at position -14 for Zm00001e018755 (*ZmICEa*, target 1) and at positions -11 and -13 for Zm00001e008118 (*ZmZOU/O11*). No substantial C to T base editing was detected at the other side of the nick (positions +1 to +3) or outside of the sgRNA binding range. The observed editing window from -7 to -14 is in overall agreement with previous work (Zong et al., 2017) despite slightly different boundaries (-9 to -15).

### Base editing of *ZmICEa* impacts stomata and plant growth

Stable transformation of the construct aiming at target 2 in *ZmICEa* (Zm00001e018755) demonstrated the predictive value of protoplast work. All 5 transformation events carried the C to T change at position -13 at target 2, which was the most frequently observed change in protoplasts (**Figure 6**). One of the 5 events contained in addition a double C to T mutation in positions -8 and -9 at target 2, which corresponded to the next most frequent modifications in protoplasts. More unexpectedly, another of the 5 events also carried a C to G mutation changing the triplet TCA (serine) to a TGA (stop codon), a modification also found in protoplasts but with a much lower frequency.

**Figure 7:**
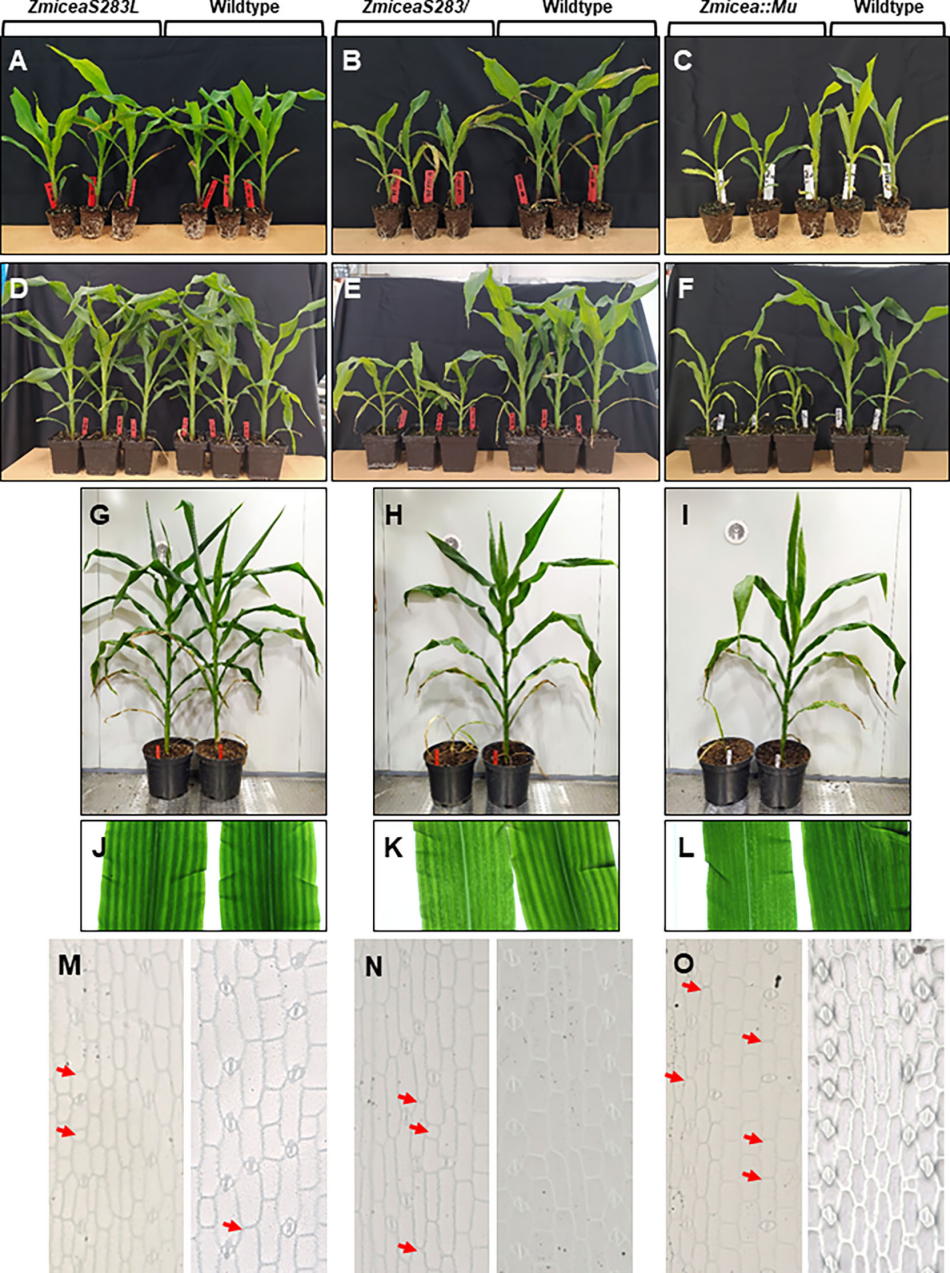
Base editing of *ZmICEa* impacts plant growth. **(A–I)** The *ZmiceaS283L* **(A, D, G, J, M)**, *ZmiceaS283/* **(B, E, H, K, N)** and *Zmicea::Mu* **(C, F, I, L, O)** mutants (T2 generation without the Cas9/sgRNA transgene, left half of the panel) and wildtype siblings (right half of the panel) were photographed 25 days after sowing (DAS, **A-C**), at 38 DAS **(D-F)** and at 66 DAS **(G-I)**. At 25 DAS the plants were removed from their pots to visually evaluate root development at the surface. **(J-L)** Observation of mutant (left) and wildtype (right) leaves on a trans-illuminator. **(M-O)** Light microscopy of leaf imprints of mutant (left) and wildtype (right) leaves. Red arrows indicate positions where stomata were expected.

To assess the phenotypic impact of the loss of the single predicted phosphorylation site in ZmICEa, the corresponding *ZmiceaS283L* mutant together with the *ZmiceaS283/* mutant carrying a stop codon in the same position, and an insertional mutant *Zmicea::Mu* were propagated in parallel (**Figure 7**). In the T1 generation, heterozygous plants carrying the respective mutations but lacking the Cas9/sgRNA transgene were selected and self-pollinated. Phenotypic characterization was carried out on homozygous T2 plantlets. At 25 days after sowing (DAS), no notable difference in plant growth was observed for the *ZmiceaS283L* mutant, whereas the *ZmiceaS283/ *and *Zmicea::Mu* mutants were smaller and had less developed root systems than wildtype siblings (**Figures 7A-C**). At 38 DAS (**Figures 7D-F**) and 66 DAS (**Figures 7G-I** and **Supplementary Figure S6**), the *ZmiceaS283L* mutant continued to grow similarly to wildtype siblings, while mutants *ZmiceaS283/* and *Zmicea::Mu* stopped growth and eventually died. Observation of leaves on a trans-illuminator revealed that mutants *ZmiceaS283/* and *Zmicea::Mu* had more transparent, paler leaves compared to wildtype and mutant *ZmiceaS283L* and presented dark green spots in the pale zones (**Figures 7J-L**). Leaf imprints indicated more frequent aberrations from the regularly spaced stomata pattern in mutants *ZmiceaS283/* and *Zmicea::Mu* than in wiltype and mutant *ZmiceaS283L* (**Figures 7M-O**). A more detailed analysis of stomata of the *Zmicea::Mu* mutant showed that they appeared small and abnormally shaped (**Supplementary Figure S7**). In many cases, even when the stomata looked relatively normal, the aperture of the pore which is formed by separation of the two guard cells, was not fully formed in the mutant (**Supplementary Figure S7**). To quantify this stomatal phenotype, indexes of normally shaped stomata, abnormally shaped stomata and meristemoids (epidermis cell number per stomata or meristemoid) on the adaxial face of the third leaf were calculated (**Supplementary Figure S7J**). *Zmicea::Mu* plants had 5.5-fold more abnormally shaped stomata, 5.5-fold fewer normally shaped stomata and 1.3-fold more meristemoids than wildtype.

## Discussion

We present here an optimized maize protoplast system and a specifically developed bioinformatics pipeline to evaluate rapidly the efficiency of CRISPR/Cas9 constructs or of novel Cas9 variants before engaging in time- and resource-consuming stable transformation.

### Protoplasts allow rapid evaluation of genome editing tools

A revisited and streamlined protocol for the preparation and transformation of maize leaf protoplasts was used to characterize the type and frequency of the modifications triggered by the CRISPR/Cas9 genome editing tool. The system showed a good repeatability between samples analyzed in two independent experiments and the comparison with the type and frequency of the modifications observed after stable transformation with some of the constructs suggested that the results of transient transformation in protoplasts were a good indicator to predict ulterior maize transformation.

As expected, the most frequently observed modifications were small indels at the cleavage site of the Cas9 enzyme 3 bp upstream of the PAM site (Chen et al., 2019; Doll et al., 2019). If most of the time the frequency of the deletions decreased both with their size and the distance of the deletion starting point from the cleavage site, in several cases specific deletions of several bases deviated from this overall pattern, for example, deletions between positions -9 and -2 in the target sequence used to compare different scaffolds (**Figure 4B**).

There are obvious limits to the analysis of genome editing events by PCR followed by NGS. PCR will only amplify events where both primer sequences remain present in the genome and even in paired-end mode, only PCR products smaller than 300 bp can be fully analyzed on an Illumina NextSeq500 platform, restricting the analysis to the vicinity of the target site and excluding the detection of deletions or insertions larger than 300 bp as well as chromosome rearrangements. Large deletions of dozens of kb (Ordon et al., 2017) and recombination between chromosomes (Schmidt et al., 2020) have been reported in Arabidopsis, although they remain considerably less frequent than small indels.

For user-friendly analysis of the NGS data, a 7-step bioinformatics pipeline combining existing programs and custom-made Python scripts was developed. A first key point was the choice of the Needleman & Wunsch algorithm with a custom scoring matrix for pairwise alignments with the reference sequence to guarantee a systematic alignment over the entire length of the reference sequence and satisfactorily handle the often important size differences between the mutated and reference sequence. The other major asset was the development of an original graphical output that resumes in an intuitive logo the nature, frequency and position of the genome modifications (**Figure 2**).

### Longer stems in certain plant sgRNA scaffolds do not improve genome editing

Cas9 is an inherently efficient nuclease leaving little room for improvements. On the other hand, the sgRNA is a biotechnological engineering product raising the question, whether the initial fusion of the crRNA and a normally trans-encoded tracrRNA into a single sgRNA molecule (Jinek et al., 2012; Cong et al., 2013) was the optimal solution to form a complex with the Cas9 and to be active in plants. For example, the two initial designs of Jinek et al. (2012), (**Supplementary Figure S3**) had quite different activity *in vitro* and underlined the importance of a minimum length of the sgRNA. The comparison of the most frequently used scaffold in plants (Shan et al., 2013) with two alternative designs with longer stems (Miao et al., 2013; Dang et al., 2015) in the maize protoplast system did not reveal any tangible differences in the efficiency of the three designs. Keeping in mind that more profound changes in the sgRNA scaffold, such as the addition of MS2 hairpins attracting transcriptional activator complexes (Chavez et al., 2016), also do not seem to notably reduce the efficiency of the system, one may conclude that the sgRNA-Cas9 interaction is rather robust to change, as long as minimum length requirements are fulfilled.

To increase the overall efficiency of genome editing in maize, other approaches may be more promising, for example the use of the *Babyboom/Wuschel* system to overcome genotype dependency (Lowe et al., 2016) and to shorten the duration of *in vitro* culture steps by the use of somatic embryogenesis (Masters et al., 2020). For genome editing of recalcitrant elite lines, trans editing also called HI editing (Kelliher et al., 2019) based on in planta transfer of the editing tool from easily transformable lab varieties also holds considerable promise (Jacquier et al., 2020). Another important consideration frequently neglected is the vectorization of Cas9. Recent studies underline the importance of optimized promoter-Cas9 and Cas9-terminator junctions for efficient genome editing (Castel et al., 2019) and report a substantial improvement by the introduction of introns in the *Cas9* coding sequence (Grutzner et al., 2021).

### Relaxed PAM increases the choice of target sites for genome editing

For the first time, the activity of two Cas9 variants, xCas9 and Cas9-NG, has been assessed in maize. The efficiency of the two enzymes recognizing relaxed PAM sites differed in maize protoplasts depending on the type of the PAM sequence. In the case of the canonical NGG PAM, the lower activity of Cas9-NG compared to xCas9 observed here, has been reported before in rice protoplasts (Zhong et al., 2019) and mammalian cells (Nishimasu et al., 2018). On the other hand, other studies report higher efficiency of Cas9-NG compared to xCas9 on NGG PAM targets in stable Arabidopsis (Ge et al., 2019) and rice transformants (Ren et al., 2019). Consequently, the relative efficiency of xCas9 and Cas9-NG in an NGG PAM context seems to vary, possibly depending on the protospacer context, the species, the vectorization (promoter, terminator) of the Cas9, and/or the method of transformation. Our study did not include a wildtype Cas9 for the same target sites, but several studies have shown that it systematically displays higher efficiencies than xCas9 and Cas9-NG on targets with NGG PAM sequence (Nishimasu et al., 2018; Ge et al., 2019; Ren et al., 2019; Zhong et al., 2019). Therefore, native Cas9 remains the system of choice for a targeted mutagenesis aiming at a single 20 nt target followed by NGG.

In the context of the non-canonical NGC PAM, the efficiencies observed here for indel induction at the target site were considerably higher for Cas9-NG than for xCas9. This observation is in agreement with other studies which consistently report higher efficiency of Cas9-NG compared to xCas9 in rice (Ren et al., 2019; Zhong et al., 2019), Arabidopsis (Ge et al., 2019) and tomato (Niu et al., 2020) with non-NGG PAMs. The efficiency observed in maize protoplasts for xCas9, at least at the tested alternative CGC PAM site, seems incompatible with routine use in stable maize transformation, despite the fact that a C in the last position of the PAM site is less favorable than a T or A (Nishimasu et al., 2018). In contrast, the frequency for Cas9-NG was more encouraging and it would be interesting to test the system to see if stable maize transformants can be generated at a reasonable rate. In conclusion, Cas9-NG but not xCas9 has the potential to expand the scope of putative targets in the maize genome not only for targeted mutagenesis but also for base or prime editing using a nickase version of the Cas9-NG backbone.

### Efficient base editing in maize

C to T base editing with the nCas9-PBE (Zong et al., 2017) in maize protoplasts gave rise to contrasting results for three target sites in *ZmICEa* (Zm00001e018755) and *ZmZOU/O11* (Zm00001e008118), two transcription factors of the bHLH family (Grimault et al., 2015; Feng and Song, 2018). In *ZmICEa*, target 1 and target 2 were edited with a high efficiency comparable to targeted mutagenesis. The edits were almost exclusively of the C to T type and limited to a window from positions -7 to -14 counting from the nicking site, which is in agreement with the -9 to -15 window reported previously (Zong et al., 2017). Furthermore, the relative frequencies of edits observed during transient expression in protoplasts had predictive value for the edits actually found in stable transformants. Stable transformation also demonstrated that rare events in protoplasts may occasionally be found in transgenic maize plants, such as a C to G mutation creating a stop codon in ZmICEa.

Unexpectedly, base editing using the same base editor and identical criteria for sgRNA design was 25 times less efficient for *ZmZOU/O11*. There is no obvious explanation for this difference, but the situation is reminiscent of targeted mutagenesis of ZmZOU/O11 with an active Cas9, which has so far proven impossible to achieve in our hands, although another study managed to obtain mutant alleles (Feng et al., 2018). One may hypothesize differences in the accessibility of ZmICEa and ZmZOU/O11 for the CRISPR/Cas9 tools, for example related to different degrees of chromatin condensation. It has been shown that chromatin de-condensation by Trichostatin A (TSA) can increase the efficiency of CRISPR/Cas9-mediated indel formation in lettuce and tobacco protoplasts (Choi et al., 2021).

The results also highlighted some current limitations of base editing, which remains limited to C and A base editors that act in a narrow window. T and G base editors are needed to complete the tool kit, while variations in the length of the linker between the nCas9 and the base editor domain can give access to other editing windows (Tan et al., 2019). Optimized prime editing fusing nCas9 to a reverse transcriptase rather than a base editor is another promising alternative to overcome present limitations (Lin et al., 2021; Xu et al., 2022).

### ZmICEa is necessary for stomatal development and plant growth

In Arabidopsis, AtICE1, the founding member of the ICE family, is a bHLH transcription factor involved in three different pathways: cold-tolerance (Chinnusamy et al., 2003), stomatal development (Kanaoka et al., 2008) and seed development (Xing et al., 2013; Denay et al., 2014). In leaves, AtICE1 and AtCRM2 form heterodimers with AtSPEECHLESS, AtMUTE and AtFAMA, three bHLH transcription factors, to regulate stomatal development (Kanaoka et al., 2008). This regulation is conserved in grasses such as Brachypodium (Raissig et al., 2016) and rice (Wu et al., 2019), although the wiring is somewhat modified (Nunes et al., 2020). These data, together with the stomatal phenotype reported in ZmZOU/O11 ectopic expression lines (Grimault et al., 2015), and the fact that *ZmiceaS283/* and *Zmicea::Mu* mutants showed reduced growth and chlorosis, led us to test whether ZmICEa (Zm00001e018755) plays a role in stomatal development. We found that in *Zmicea::Mu* mutants, even when the stomata looked relatively normal, the aperture of the pore was not fully formed, a phenotype which is likely to restrict gas exchange potentially leading to the chlorotic leaf phenotype. We also found abnormally shaped stomata similar to those observed in the *Osfama-1* mutant (Liu et al., 2009). Our data suggest that, contrary to AtICE1 which, redundantly with AtSCRM2, is necessary throughout stomatal development, ZmICEa could be specifically and non-redundantly be required during the last steps of stomatal differentiation. It would be interesting to test the physical interactions of ZmFAMA with ZmICEa in order to see whether this apparently specific late role reflects a specific protein binding affinity.

The absence of a strong stomatal phenotype in the *ZmiceaS283L* mutant, where base editing prevents the predicted phosphorylation of the serine residue, may be explained either by the fact that this phosphorylation is not indispensable for ZmICEa to fulfill its role in stomatal development, or by a stabilization of ZmICEa in the absence of phosphorylation, leading to a gain-of-function rather than loss-of-function phenotype similarly to what has been reported for the semi-dominant *Atice1-1/AtScrm-D* allele (Chinnusamy et al., 2003).

## Data availability statement

The data for this study have been deposited in the European Nucleotide Archive (ENA) at EMBL-EBI under accession number PRJEB56234 (https://www.ebi.ac.uk/ena/browser/view/PRJEB56234).

## Author contributions

ND-F, AG, TW, and PR designed the research; YF, NMAJ, EM, CR, and JL-M performed research; LG and JJ contributed new computational tools; YF, NMAJ, LG, JJ, JL-M, TW, and PR analyzed data; and YF and PR wrote the paper. All authors contributed to the article and approved the submitted version.

## Funding

The work was financed in part by the Investissement d’Avenir program of the French National Agency of Research for the project GENIUS (ANR-11-BTBR-0001_GENIUS). YF was supported by a CIFRE fellowship of the ANRT (grant N°2018/0480).

## Acknowledgments

We thank Justin Berger, Alexis Lacroix and Patrice Bolland for maize culture, Hervé Leyral and Isabelle Desbouchages for media preparation, Claire Lionnet for assistance with epifluorescent microscopy, Ghislaine Gendrot and Kathy Gallay for maize transformation, and Antoine Heurtel, Marie Martelat and Hadrien Guichard for initial developments of the bioinformatics pipeline. Benjamin Gillet and Sandrine Hughes (ENS de Lyon) of the IGFL sequencing platform (PSI) are acknowledged for expert advice, library preparation and Illumina runs, and Stefan Scholten (University Göttingen) for tricks in maize protoplast isolation. Credits are attributed to Bakunetsu Kaito, Yohann Berger, Julie Ko, Smalllike, N.Style and IYIKON for their vectorial images under CC BY 3.0 license, (https://thenounproject.com/) used and modified in **Figure 1A**.

## Conflict of interest

YF and AG were employed by MAS Seeds, NMAJ is presently employed by Limagrain Europe, TW has currently a collaborative research project with Limagrain Europe, and PR is a member of the operational directorate of the PlantAlliance consortium.

The remaining authors declare that the research was conducted in the absence of any commercial or financial relationships that could be construed as a potential conflict of interest.

## Publisher’s note

All claims expressed in this article are solely those of the authors and do not necessarily represent those of their affiliated organizations, or those of the publisher, the editors and the reviewers. Any product that may be evaluated in this article, or claim that may be made by its manufacturer, is not guaranteed or endorsed by the publisher.
