# Supplementary material for: Evaluation of genome and base editing tools in maize protoplasts
Source: Front Plant Sci. 2022 Nov 28;13:1010030. doi: 10.3389/fpls.2022.1010030 (PMC9744195; doi:10.3389/fpls.2022.1010030)
Supplement: Supplementary file 1 [file Presentation_1.pdf]

Supplementary Figure S1

A

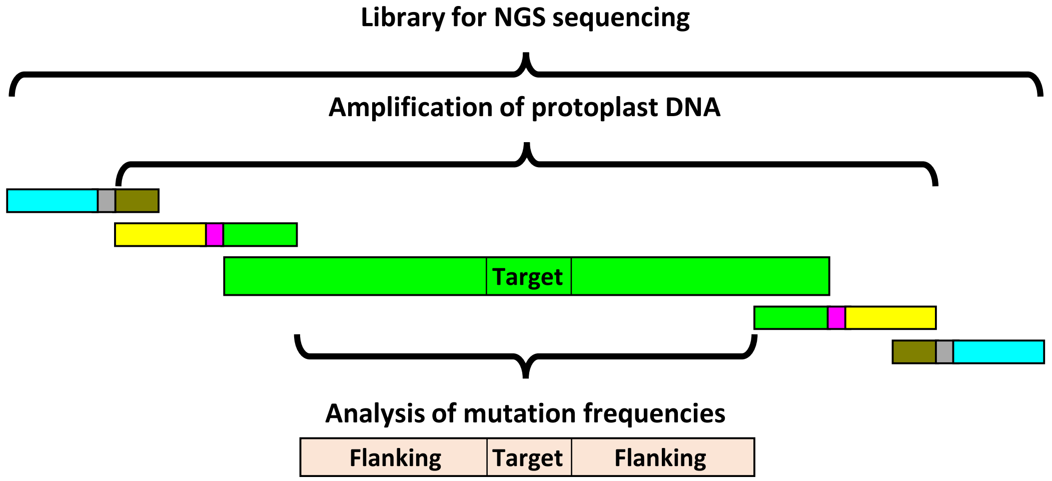

B

```
5'-AATGATACGGCGACCACCGAGATCTACAC[i5]TCGTCGGCAGCGTC-3'
                                  5'-TCGTCGGCAGCGTCAGATGTGTATAAGAGACAGNNNNNAGATAAGGGTAAGGAGCAGCAGCAG-3'

      3'-GGACCTGCTATATGACGGTAGCNNNNNGACAGAGAATATGTGTAGAGGCTCGGGTGCTCTG-5'
                                                   3'-GGCTCGGGTGCTCTG[i7]TAGAGCATACGGCAGAAGACGAAC-5'
```

Supplementary Figure S1 | Library construction and NGS analysis
A) Scheme depicting the initial amplification of protoplast DNA with primers composed of a gene specific part (green), a 5 nt tag (purple) and a tail (yellow) with homology to the adapter used for library construction. The adapter is composed of the homologous part (army green), an index (grey) and a tail (blue). The analysis of mutation frequencies excluded the primer regions and was separated for the 20 nt target site and upstream and downstream flanking regions. B) DNA sequences of the oligonucleotides used for the library construction of Zm00001e008508. [i5] and [i7] are fixed 5 nt or 7 nt indexes used to identify libraries after multiplexing during the sequencing run. NNNNN is a 5 nt tag with a random sequence during the synthesis of the oligonucleotide allowing to discriminate between PCR products.

Supplementary Figure S2

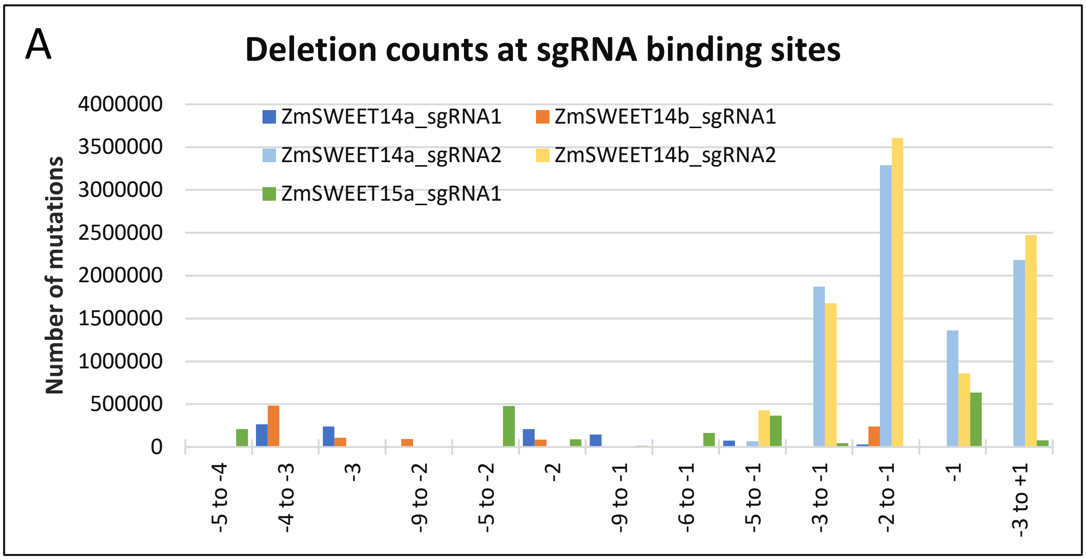

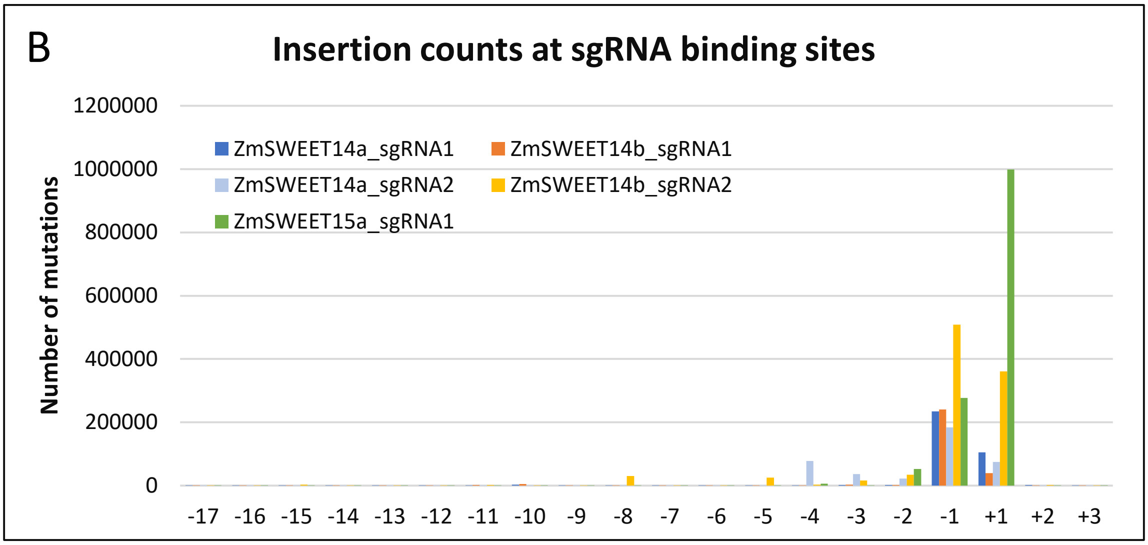

Supplementary Figure S2 | (A, B) Graphs indicating the deletion (A) and insertion (B) counts of mutations for selected deletions (A) and insertions at all positions of the target sequences (B) in Zm00001e011125 (*ZmSWEET14a*), Zm00001e021494 (*ZmSWEET14b*) and Zm00001e022582 (*ZmSWEET15a*).

Supplementary Figure S3

A

```
Jinek-a                             GUUUUAGAGCUA-----GAAA-----UAGCAAGUUAAAAUAAGGCUAGUCCG------------------GG
Jinek-b                             GUUUUAGAGCUA-----GAAA-----UAGCAAGUUAAAAUAAGG--------------------------GG
Cong                                GUUUUAGAGCUA-----GAAA-----UAGCAAGUUAAAAUAAGGCUAGUCCG
Shan        AUGAUGAAGAUUCAGGGUUCGUUUUAGAGCUA-----GAAA-----UAGCAAGUUAAAAUAAGGCUAGUCCGUUAUCAACUUGAAAAAGUGGCACCGAGUCGGUGC
Miao        AUGAUGAAGAUUCAGGGUUCGUUUUAGAGCUAUGCU-GAAA-AGCAUAGCAAGUUAAAAUAAGGCUAGUCCGUUAUCAACUUGAAAAAGUGGCACCGAGUCGGUGC
Dang        AUGAUGAAGAUUCAGGGUUCGUUUCAGAGCUAUGCUGGAAACAGCAUAGCAAGUUGAAAUAAGGCUAGUCCGUUAUCAACUUGAAAAAGUGGCACCGAGUCGGUGC
```

B

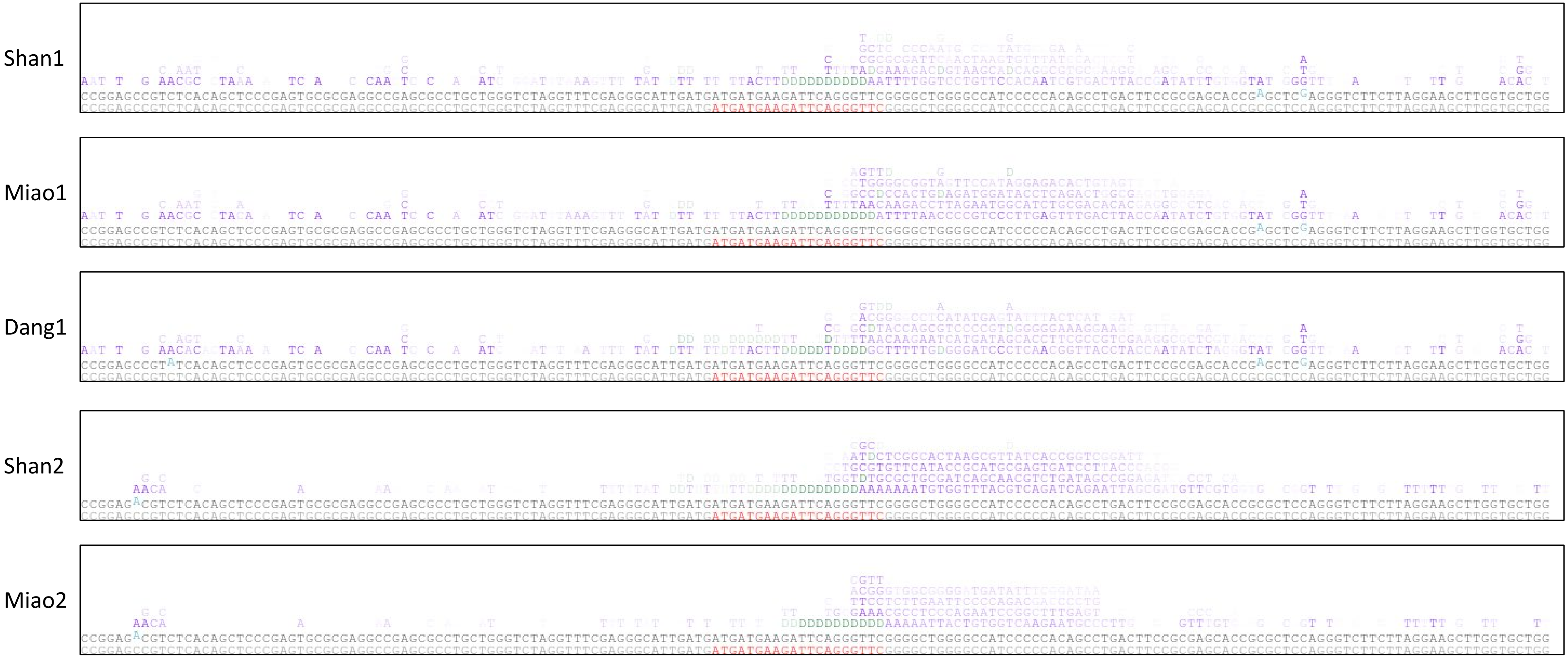

Supplementary Figure S3 | Targeted mutagenesis with different scaffolds. A) Sequence alignment of first generation scaffolds (Jinek-a, Jinek-b, Cong) and the three experimentally tested scaffolds Shan, Miao and Dang. For the latter three, the entire sgRNAs targeting Zm00001e008508 are shown including the 20 nucleotides complementary to the target sequence (highlighted in yellow). Mismatches are highlighted in red. B) Logos of the Zm00001e008508 fragment amplified for NGS after protoplast mutagenesis with the three scaffolds. Shan1 and Shan2, as well as Miao1 and Miao2, are biological replicates.

Supplementary Figure S4

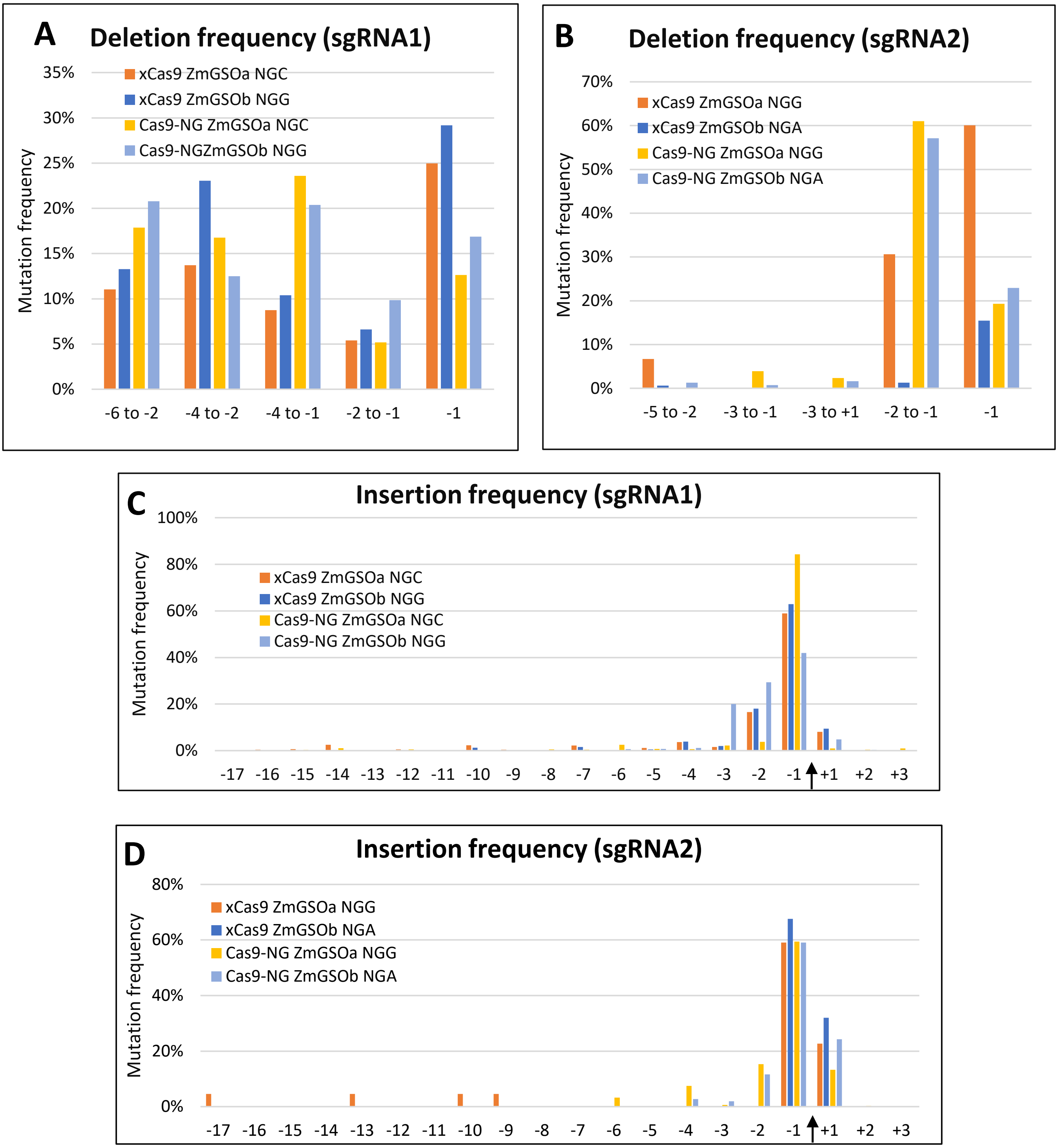

Supplementary Figure S4 | xCas9 and Cas9-NG-mediated indel frequencies. (A to D) Graphs indicating the mutation frequencies for selected deletions (A, B) and insertions at all positions of the 20 nt target sites (C, D) of sgRNA1 and sgRNA2 in *ZmGSOa* (NGC PAM for sgRNA1) and *ZmGSOb* (NGA PAM for sgRNA2) generated by xCas9 and Cas9-NG. The Cas9 cleavage site is indicated by an arrow.

Supplementary Figure S5

Base editing Zm00001e018755 (*ZmICEa*, target 1)

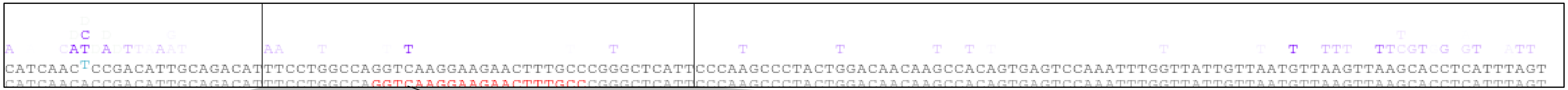

Position	-27	-26	-25	-24	-23	-22	-21	-20	-19	-18	-17	-16	-15	-14	-13	-12	-11	-10	-9	-8
Reference	T	T	C	C	T	G	G	C	C	A	G	G	T	C	A	A	G	G	A	A
G	220	240	326	1 324	899	0	0	1 764	2 817	3 221	0	0	2 068	2 443	2 831	3 659	0	0	2 461	5 247
A	30 651	23 953	4 507	2 098	3 433	3 634	8 452	2 882	4 094	0	1 586	2 176	3 352	2 802	0	0	1 987	1 560	0	0
T	0	0	2 558	2 690	0	20 077	10 429	4 503	3 869	3 830	6 527	14 695	0	463 657	12 397	10 499	9 447	11 635	8 391	6 522
C	2 551	1 950	0	0	5 941	2 641	6 578	0	0	1 321	1 944	3 136	4 887	0	1 302	1 116	1 491	2 405	740	972
Mismatch	33 422	26 143	7 391	6 112	10 273	26 352	25 459	9 149	10 780	8 372	10 057	20 007	10 307	468 902	16 530	15 274	12 925	15 600	11 592	12 741
Insertions	10	2	127	1	1	1	2	4	3	4	17	7	1	8	14	2	4	4	2	0
Deletions	1 028	2	1	294	8	1	1 231	21	1 167	4	147	591	1	26	283	324	6	341	14	307

Position	-7	-6	-5	-4	-3	-2	-1	+1	+2	+3	+4	+5	+6	+7	+8	+9	+10	+11	+12	+13
Reference	G	A	A	C	T	T	T	G	C	C	C	G	G	G	C	T	C	A	T	T
G	0	1 944	2 212	1 100	629	454	378	0	5 663	3 325	3 396	0	0	0	571	1 952	3 111	1 981	471	245
A	1 469	0	0	2 189	7 959	3 358	2 352	2 942	5 798	5 168	3 315	3 911	1 995	5 415	5 546	1 492	6 504	0	3 345	2 405
T	7 406	9 167	12 369	1 915	0	0	0	9 314	14 503	3 949	4 419	4 856	30 855	6 629	3 119	0	3 072	5 194	0	0
C	2 694	3 261	1 709	0	3 525	2 622	4 126	1 794	0	0	0	3 396	2 437	1 579	0	7 608	0	2 071	4 421	6 788
Mismatch	11 569	14 372	16 290	5 204	12 113	6 434	6 856	14 050	25 964	12 442	11 130	12 163	35 287	13 623	9 236	11 052	12 687	9 246	8 237	9 438
Insertions	4	9	1	3	20	5	5	4	42	1	2	23	4	1	3	2	6	5	165	19
Deletions	5	33	85	0	13	1 223	39	120	1 297	1	6	1 065	3	2	6	8	28	45	339	1

Base editing Zm00001e018755 (*ZmICEa*, target 2)

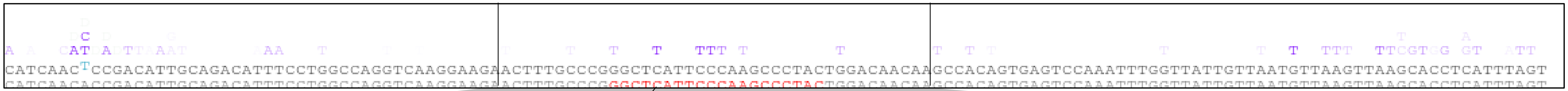

Position	-27	-26	-25	-24	-23	-22	-21	-20	-19	-18	-17	-16	-15	-14	-13	-12	-11	-10	-9	-8
Reference	A	C	T	T	T	G	C	C	C	G	G	G	C	T	C	A	T	T	C	C
G	2 407	1 589	773	554	462	0	8 250	4 403	5 502	0	0	0	729	2 424	7 233	2 085	504	258	4 245	6 190
A	0	2 792	9 427	4 361	3 913	3 315	7 144	6 389	4 104	4 394	2 283	6 194	7 461	2 123	8 826	0	4 677	2 919	2 977	7 773
T	17 008	2 188	0	0	0	11 607	20 058	4 576	4 981	5 702	36 640	7 861	13 681	0	2 045 082	7 068	0	0	301 709	145 004
C	1 917	0	3 944	2 887	4 466	2 109	0	0	0	3 903	2 763	1 818	0	8 214	0	2 046	4 826	7 348	0	0
Mismatch	21 332	6 569	14 144	7 802	8 841	17 031	35 452	15 368	14 587	13 999	41 686	15 873	21 871	12 761	2 061 141	11 199	10 007	10 525	308 931	158 967
Insertions	4	4	27	17	14	4	36	0	1	26	6	6	13	19	10	15	194	37	27	2
Deletions	96	0	18	1 343	44	0	149	1 358	66	6	1 041	50	22	29	23	263	57	383	2	1 710

Position	-7	-6	-5	-4	-3	-2	-1	+1	+2	+3	+4	+5	+6	+7	+8	+9	+10	+11	+12	+13
Reference	C	A	A	G	C	C	C	T	A	C	T	G	G	A	C	A	A	C	A	A
G	5 232	4 141	5 334	0	1 780	3 179	1 882	967	2 449	5 163	1 109	0	0	6 247	6 488	3 388	3 280	4 805	2 782	3 763
A	4 924	0	0	2 192	2 182	7 922	9 281	2 601	0	6 745	2 926	3 842	4 400	0	1 692	0	0	9 879	0	0
T	65 650	8 349	36 810	9 050	3 045	3 758	2 536	0	7 233	2 061	0	43 664	8 361	7 124	5 210	9 102	9 370	3 129	14 112	9 606
C	0	2 606	1 830	4 023	0	0	0	4 752	1 327	0	5 273	4 926	2 954	1 429	0	2 827	2 472	0	3 029	2 959
Mismatch	75 806	15 096	43 974	15 265	7 007	14 859	13 699	8 320	11 009	13 969	9 308	52 432	15 715	14 800	13 390	15 317	15 122	17 813	19 923	16 328
Insertions	6	18	3	12	18	2	8	19	2	5	4	2	6	11	3	18	8	6	4	5
Deletions	219	24	293	17	142	1 939	3	212	246	21	46	1 466	5	248	334	265	8	245	225	2

Base editing Zm00001e008118 (*ZmZOU/O11*)

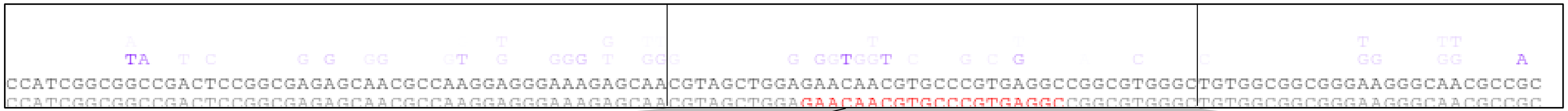

Position	-27	-26	-25	-24	-23	-22	-21	-20	-19	-18	-17	-16	-15	-14	-13	-12	-11	-10	-9	-8
Reference	C	G	T	A	G	C	T	G	G	A	G	A	A	C	A	A	C	G	T	G
G	16 334	0	896	10 666	0	4 132	3 320	0	0	21 665	0	25 840	18 608	2 153	20 088	19 087	5 220	0	1 590	0
A	2 377	9 048	1 950	0	4 648	3 196	8 565	4 900	4 343	0	4 633	0	0	1 830	0	0	1 644	10 957	5 024	6 894
T	11 138	7 493	0	9 626	7 774	6 515	0	4 887	7 814	11 700	7 394	9 028	8 389	79 016	10 488	16 109	48 603	9 279	0	4 208
C	0	3 544	10 182	2 253	1 391	0	13 231	604	818	1 081	1 316	1 637	1 396	0	1 594	4 579	0	2 509	16 111	2 384
Mismatch	29 849	20 085	13 028	22 545	13 813	13 843	25 116	10 391	12 975	34 446	13 343	36 505	28 393	82 999	32 170	39 775	55 467	22 745	22 725	13 486
Insertions	9	13	9	8	12	33	26	10	12	45	25	33	9	14	53	33	19	44	51	44
Deletions	15	59	49	37	55	7	66	138	5 186	15	299	81	146	16	260	197	24	53	96	38

Position	-7	-6	-5	-4	-3	-2	-1	+1	+2	+3	+4	+5	+6	+7	+8	+9	+10	+11	+12	+13
Reference	C	C	C	G	T	G	A	G	G	C	C	G	G	C	G	T	G	G	G	C
G	2 055	12 803	17 888	0	4 924	0	34 510	0	0	6 285	4 561	0	0	5 436	0	6 324	0	0	0	9 582
A	2 181	2 943	3 607	8 875	4 599	4 678	0	5 207	5 380	1 967	3 023	13 614	6 478	1 625	8 068	4 313	6 884	7 025	5 389	2 311
T	8 864	8 164	11 821	8 847	0	8 727	13 888	2 237	2 131	9 583	10 705	7 299	2 791	12 693	7 259	0	9 431	4 764	4 156	9 807
C	0	0	0	614	16 214	715	3 955	7 140	2 633	0	0	897	822	0	1 924	19 222	590	396	802	0
Mismatch	13 100	23 910	33 316	18 336	25 737	14 120	52 353	14 584	10 144	17 835	18 289	21 810	10 091	19 754	17 251	29 859	16 905	12 185	10 347	21 700
Insertions	500	6	12	31	37	32	38	21	27	27	21	34	11	21	24	33	32	26	32	10
Deletions	45	2 151	53	110	98	531	67	299	29	372	21	148	51	46	61	172	9 415	41	16	66

Supplementary Figure S5 | Base editing. For each target site are presented on the top the logo of the fragment amplified for NGS and on the bottom a table indicating the number and type of mutations for every position of the 20 nt target sequence (red) and the 10 nt upstream and downstream. In the table the PAM site is in green and positions of selected bases refer to the nCas9 nick site. The values of the table are coloured by a heat map with red for highest values and green for lowest values.

Supplementary Figure S6

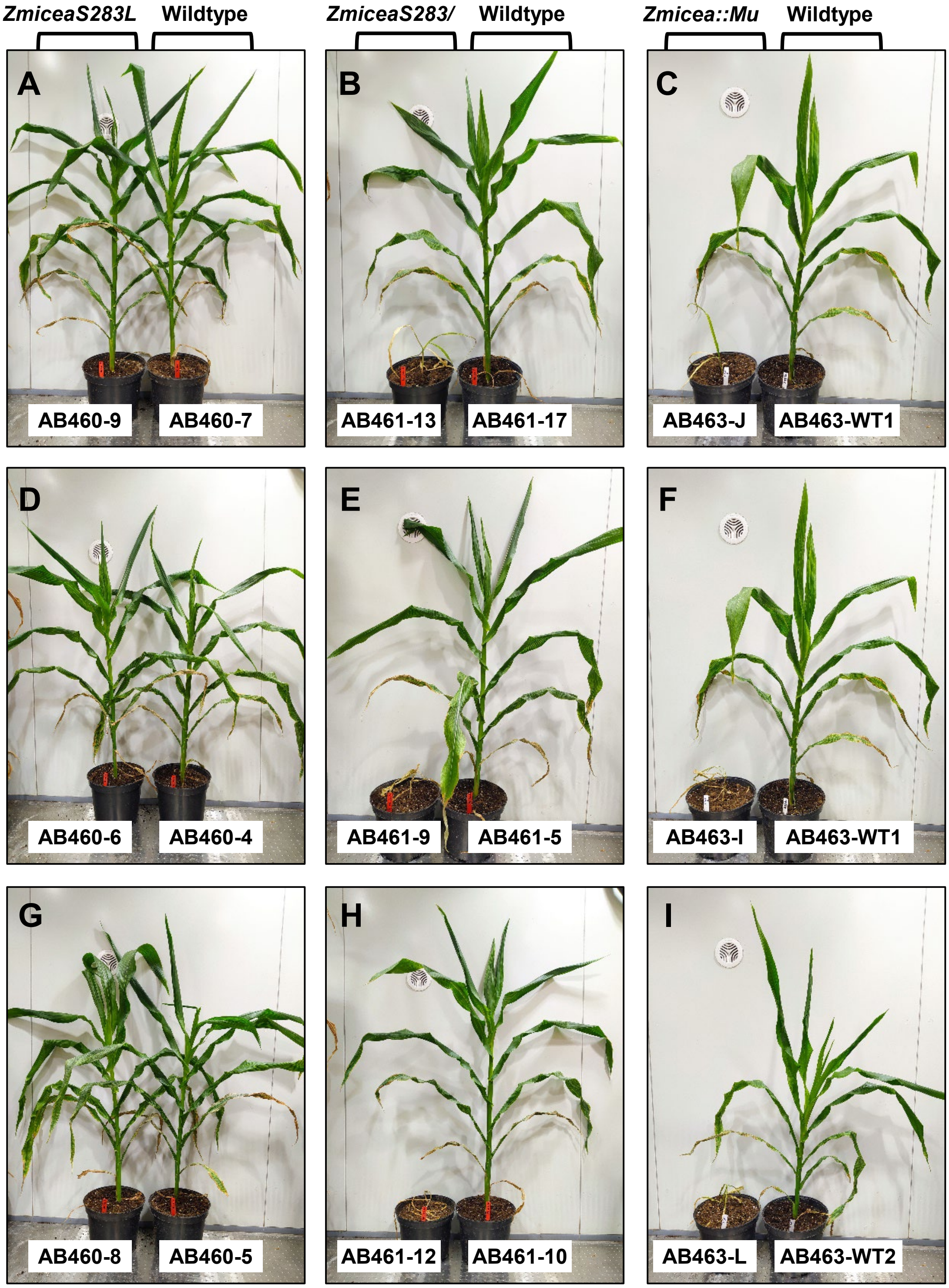

Supplementary Figure S6 | Base editing of *ZmICEa* impacts plant growth (A-I) The *ZmiceaS283L* (A, D, G), *ZmiceaS283/* (B, E, H) and *Zmicea::Mu* (C, F, I) mutants (T2 generation without the Cas9/sgRNA transgene, left half of the panel) and wildtype siblings (right half of the panel) were photographed 66 days after sowing (DAS). Panels (A, B, C) are identical to panels (G, H, I) of Fig. 7.

Supplementary Figure S7

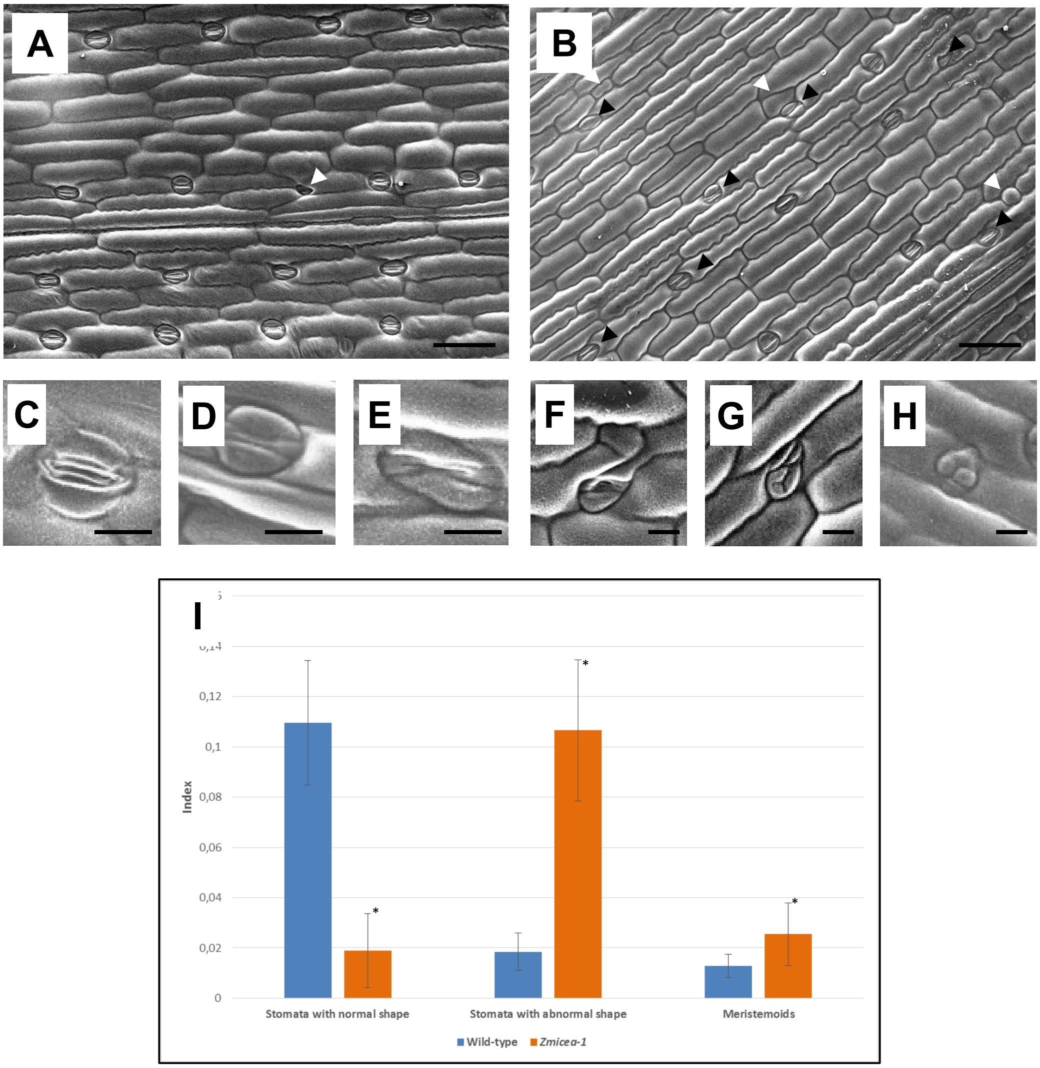

Supplementary Figure S7 | Stomatal development in the *Zmicea::Mu* mutant (A-B) 11 days after sowing, *Zmicea::Mu* mutants (B) produce a high proportion of undeveloped meristemoids and abnormally shaped stomata compared to wild-type siblings (A). (C-H) Zoom of normal shaped stomata in a wildtype plant (C) and abnormal stomata in *Zmicea::Mu* mutants (D-H). Black head arrows indicate abnormal shaped stomata and white head arrows indicate meristemoids. Scale bars: 50 µm (C-E) and 10 µm (F-H). (I) Indexes of normal shaped stomata, abnormal shaped stomata and meristemoids on the adaxial face of the third leaf were calculated at 11 days after sowing using environmental transmission electronic microscopy images. Error bars correspond to the standard deviation calculated from all measurements. Three different areas were measured for four homozygous *Zmicea::Mu* and three wild-type leaves. A Mann-Whitney U-test was applied * = $P < 0.001$.

Supplementary Table S1 | Original vectors used in this study

Vector name	Vector type	Promoter driving Cas9	Cas9	Promoter driving sgRNA	sgRNA scaffold
L1537	Integrative	prZmUBI (synthetic)	Cas9 (codon optimised for rice)	OsU3	long (Miao)
L1608	Small	NA	No	OsU3	short (Shan)
L1609	Integrative	prZmUBI (synthetic)	Cas9 (codon optimised for rice)	OsU3	short (Shan)
L1611	Small	NA	No	TaU6	short (Shan)
L1944	Integrative	prZmUBI (synthetic)	No	OsU3	short (Shan)
L1945	Integrative	prZmUBI (synthetic)	CDA-nCas9-UGI (codon optimised rice)	OsU3	short (Shan)
L1966	Integrative	prZmUBI (synthetic)	xCas9 (codon optimised for maize)	OsU3	short (Shan)
L2023	Integrative	prZmUBI (synthetic)	Cas9 (codon optimised for rice)	ZmU6 (C1-long)	short (Shan)
L2008	Integrative	prZmUBI (synthetic)	Cas9-NG (codon optimised for maize)	OsU3	short (Shan)

Supplementary Table S2 | Maize transformation media

	LS inf	LSA (1 week)	LSD5 (2 weeks)	LSD10+ (3 weeks)	LSD10++ (3 weeks)	LSZ1 (2 to 3 weeks)	LSZ2 (2 to 3 weeks)	RM (1 to 2 weeks
MS salts [g/L][1]	4,44	4,44	4,44	4,44	4,44	4,44	4,44	
MS salts [g/L][2]								4,44
Sucrose [g/L]	68,5	20	20	20	60	40	20	20
Glucose [g/L]	36	10						
L-proline [g/L]		0,7	0,7	0,7	0,7	0,7	0,7	0,7
MES [g/L]		0,5	0,5	0,5	0,5	0,5	0,5	0,5
L-Cystine [g/L]		0,4						
1000 x Vitamin mix [mL][3]	1	1	1	1	1	1	1	
2,4 D [mg/L]	1,5	1,5		0,5	0,5			
Dicamba [mg/L]			5					
Acetosyringone [µM]	100	100						
$CuSO_4$ [µM]		5						
$AgNO_3$ [µM]		5	10					
Cefotaxime [mg/L]			250	250	250	250	100	50
Glufosinate [mg/L]			5	10	10	5	4	2
Ancymidole [mg/L]						0,25		
Kinetin [mg/L]						0,5		
Purified agar [g/L]		8	8	8	8			
Gelzan [g/L]						2,3	2,3	2,3
pH	5,2 (KOH)	5,8 (KOH)	5,8 (KOH)	5,8 (KOH)	5,8 (KOH)	5,8 (KOH)	5,8 (KOH)	5,8 (KOH)

[1] Murashige and Skoog salts (reference M 6899 Sigma)

[2] Murashige and Skoog salts (reference M 5519 Sigma)

[3] 1000 x Vitamin mix: 500 mg/L Nicotinic acid (vitamin B3), 500 mg/L Pyridoxine (Vitamin B6) and 600 mg/L Thiamine (Vitamine B1)

Supplementary Table S3 | CRISPR/Cas9 target sites, guides and primers used in this study

Experimental code	Plasmid	Experiment	Material	Target gene	GeneID v5	Localisation	Modification	Target sequence with PAM[1,2,3]	Forward primer name	Forward primer sequence (no tail)	Reverse primer name	Reverse primer sequence (no tail)	Expected size (no tail)	PCR conditions on transformed protoplast DNA
NP06	L1750	Scaffold	Leaf protoplasts	ZmKAK1	Zm00001e008508	chr2	Targeted mutagenesis	ATGATGAAGATTCAGGGTTC*GGG*	KAK1-BEd-3F	AGATAAGGGTAAGGAG CAGCAG	KAK1-BEd-3R	CGATGGCAGTATATCG TCCAGG	217 bp	Single PCR primers with tail, Phusion 35 cycles, hybridation 65°C 30sec, elongation 15sec
NP07	L1972	Scaffold	Leaf protoplasts	ZmKAK1	Zm00001e008508	chr2	Targeted mutagenesis						217 bp	
NP08	L1977	Base editing	Leaf protoplasts	ZmICEa	Zm00001e018755	chr3	Base editing	GGTCAAGGAAGAACTTTGCC*CGG*	ICE-BEd-3F	GGACCAACATCAGCTA GCTTCAACC	ICE-BEd-3R	GCAGGCTTAAGTAGCA ATGTATCACACA	196 bp	Single PCR primers with tail, Phusion 35 cycles, hybridation 65°C 30sec, elongation 15sec
NP09	L1978	Base editing	Leaf protoplasts	ZmICEa	Zm00001e018755	chr3	Base editing	GGCTCATTCCCAAGCCCTAC*TGG*					196 bp	
NP10	L1979	Base editing	Leaf protoplasts	ZmZOU	Zm00001e008118	chr2	Base editing	GAACAACGTGCCCGTGAGGC*CGG*	ZOU-BEd-1F	TGGGTCGTTCAAGGCT GCCAGA	ZOU-BEd-1R	TGGTCCACATCCTCCA CCGCG	159 bp	PCR1 primers no tail 15 cycles, GoTaq, 55°C hyb, 30sec elongation. Dilution 1/10. PCR2 primers with tail 23 cycles with Phusion hotstart, 65°C hyb
NP11	L1992	xCas9	Leaf protoplasts	ZmGSOa ZmGSOb	Zm00001e035023 Zm00001e010407	chr7 chr2	Targeted mutagenesis	CGAGTTCACCGGAGCAATCC*CGG*	ZmGSO-BEd-G2-1F	SAGCCACAACCGCCTG TC	ZmGSO-Bed-G2-1R	GCACTGTTCCATTGAT CTGG	173 bp	PCR1 primers no tail 15 cycles, Phusion Hotstart, 55°C, 15 sec elongation. Dilution 1/10. PCR2 primers with tail 23 cycles, Phusion, 65°C.
NP12	L1991	xCas9	Leaf protoplasts	ZmGSOa ZmGSOb	Zm00001e035023 Zm00001e010407	chr7 chr2	Targeted mutagenesis	ATCACAACAAGCTCACCGGT*CGG*	ZmGSO-BEd-G1-1F	TSCTCAACAACAACAG CCTCTC	ZmGSO-BEd-G1-1R	GRGAACTGGTTCTCGT ACAGGT	166 bp	PCR1 primers no tail 15 cycles, Phusion Hotstart, 55°C, 15 sec elongation. Dilution 1/10. PCR2 primers with tail 23 cycles, Phusion, 65°C.
NP13	L2017	Cas9-NG	Leaf protoplasts	ZmGSOa ZmGSOb	Zm00001e035023 Zm00001e010407	chr7 chr2	Targeted mutagenesis	ATCACAACAAGCTCACCGGT*CGG*	ZmGSO-BEd-G1-1F	TSCTCAACAACAACAG CCTCTC	ZmGSO-BEd-G1-1R	GRGAACTGGTTCTCGT ACAGGT	166 bp	PCR1 primers no tail 15 cycles, Phusion Hotstart, 55°C, 15 sec elongation. Dilution 1/10. PCR2 primers with tail 23 cycles, Phusion, 65°C.
NP14	L2018	Cas9-NG	Leaf protoplasts	ZmGSOa ZmGSOb	Zm00001e035023 Zm00001e010407	chr7 chr2	Targeted mutagenesis	CGAGTTCACCGGAGCAATCC*CGG*	ZmGSO-BEd-G2-1F	SAGCCACAACCGCCTG TC	ZmGSO-Bed-G2-1R	GCACTGTTCCATTGAT CTGG	173 bp	PCR1 primers no tail 15 cycles, Phusion Hotstart, 55°C, 15 sec elongation. Dilution 1/10. PCR2 primers with tail 23 cycles, Phusion, 65°C.
NP15	L1750	Scaffold	Leaf protoplasts	ZmKAK1	Zm00001e008508	chr2	Targeted mutagenesis	ATGATGAAGATTCAGGGTTC*GGG*	KAK1-BEd-3F	AGATAAGGGTAAGGAG CAGCAG	KAK1-BEd-3R	CGATGGCAGTATATCG TCCAGG	217 bp	Single PCR primers with tail, Phusion 35 cycles, hybridation 65°C 30sec, elongation 15sec
NP16	L1972	Scaffold	Leaf protoplasts	ZmKAK1	Zm00001e008508	chr2	Targeted mutagenesis	ATGATGAAGATTCAGGGTTC*GGG*	KAK1-BEd-3F	AGATAAGGGTAAGGAG CAGCAG	KAK1-BEd-3R	CGATGGCAGTATATCG TCCAGG	217 bp	Single PCR primers with tail, Phusion 35 cycles, hybridation 65°C 30sec, elongation 15sec
NP17	L2009	Scaffold	Leaf protoplasts	ZmKAK1	Zm00001e008508	chr2	Targeted mutagenesis	ATGATGAAGATTCAGGGTTC*GGG*	KAK1-BEd-3F	AGATAAGGGTAAGGAG CAGCAG	KAK1-BEd-3R	CGATGGCAGTATATCG TCCAGG	217 bp	Single PCR primers with tail, Phusion 35 cycles, hybridation 65°C 30sec, elongation 15sec
NP22	L1986	Correlation stable transformation	Leaf protoplasts	ZmSweet14a (2 sites)	Zm00001e011125	chr2	Targeted mutagenesis	ACTCCTCAACGTGGGCGTGTT*CGG* GACGAAGACGCTGACGGAGA*AGG*	NGS_SWT14a-b_1F	CAGCTGTTCACGGCCA AGAT	NGS_SWT14a_3R	GCGCTTCTGAAATTGA GGACTG	199 bp	Single PCR primers with tail, Phusion 35 cycles, hybridation 60°C 20sec, elongation 20sec.
NP23	L1986	Correlation stable transformation	Leaf protoplasts	ZmSweet14b (2 sites)	Zm00001e021494	chr4	Targeted mutagenesis	ACTCCTCAACGTGGGCGTGTT*CGG* GACGAAGACGCTGACGGAGA*AGG*	NGS_SWT14a-b_1F	CAGCTGTTCACGGCCA AGAT	NGS_SWT14b_2R	ATATGCATGGTGTTGG GTAGTGTA	194 bp	Single PCR primers with tail, Phusion 35 cycles, hybridation 60°C 20sec, elongation 20sec.
NP24	L1987	Correlation stable transformation	Leaf protoplasts	ZmSweet15a (2 sites)	Zm00001e022582	chr4	Targeted mutagenesis	GTACCTGGTGTACGCGCCCA*AGG* AAGACGAAGCCCAGCACGTT*GGG*	NGS_SWT15a_g1_1F	GCGTCGTCGAGACCGT GTA	NGS_SWT15a_g1_1R	GTCGGAGAGAAGCATG GTGAC	140 bp	Single PCR primers with tail, Phusion 35 cycles, hybridation 60°C 20sec, elongation 20sec.

[1] PAM sequence is in italics and underlined

[2] Red C at the 5'-end were suboptimal for transcription by a U3 promoter

[3] Red A at the 5'-end were added by hand to fit U3 promoter requirements and are not present in the genomic sequence

Supplementary Table S4 | Benchmarking of bioinformatics tools

Tool	Interface	Read preprocessing	Alignment	Output	Reference
CRISPR-proto-maize	Command-line	Pear<br>Fastq-MCF	Needleman & Wunsch	Logo and tables	this study
Hi-TOM	Web interface		bwa-mem	Tables	Liu et al., 2018
CRISPR-DAV	Command line	Prinseq<br>FLASh	bwa and ABRA	Plots, alignments and tables	Wang et al., 2017
CRISPR-GA	Web interface	Fastx-toolkit	Blat	Plots and tables	Güell et al., 2014
CRISPResso	Web interface and CLI	Trimmomatic<br>FLASh	Needleman & Wunsch	Plots and tables	Pinello et al., 2016
BATCH-GE	Command-line	Fastx-toolkit<br>Picard tools<br>bbmap	bwa-mem	Tables, UCSC genome browser	Boel et al., 2016
Cas-analyser	Client-side web interface	Fastq-join	Needleman & Wunsch	Barplots and tables	Park et al., 2017

Supplementary Table S5 | Metrics of NGS analysis

Experiment type	Scaffold Short (Shan1)	Scaffold Long (Miao1)	Scaffold Third (Dong1)	Scaffold Short (Shan2)	Scaffold Long (Miao2)	Base editing	Base editing	Base editing	xCas9	xCas9	xCas9	xCas9	Cas9-NG	Cas9-NG	Cas9-NG	Cas9-NG	Correlation stable transformation	Correlation stable transformation	Correlation stable transformation
Experimental code	NP15	NP16	NP17	NP06	NP07	NP08	NP09	NP10	NP11_type1_GSOa	NP11_type2_GSOb	NP12_type1_GSOa	NP12_type2_GSOb	NP13_type1_GSOa	NP13_type2_GSOb	NP14_type1_GSOa	NP14_type2_GSOb	NP22	NP23	NP24
Gene targeted	ZmKAK1	ZmKAK1	ZmKAK1	ZmKAK1	ZmKAK1	ZmICEa	ZmICEa	ZmZOU	ZmGSOa ZmGSOb	ZmGSOa ZmGSOb	ZmGSOa ZmGSOb	ZmGSOa ZmGSOb	ZmGSOa ZmGSOb	ZmGSOa ZmGSOb	ZmGSOa ZmGSOb	ZmGSOa ZmGSOb	ZmSweet14a (2 sites)	ZmSweet14b (2 sites)	ZmSweet15a
Plasmid	L1750	L1972	L2009	L1750	L1972	L1977	L1978	L1979	L1992	L1992	L1991	L1991	L2017	L2017	L2018	L2018	L1986	L1986	L1987
Target including primers	217 bp	217 bp	217 bp	217 bp	217 bp	196 bp	196 bp	159 bp	173 bp	173 bp	166 bp	166 bp	166 bp	166 bp	173 bp	173 bp	199 bp	194 bp	140 bp
Target excluding primers	170 bp	170 bp	170 bp	170 bp	170 bp	143 bp	143 bp	116 bp	135 bp	135 bp	122 bp	122 bp	122 bp	122 bp	135 bp	135 bp	157 bp	150 bp	100 bp
CRISPR range [length]	20 bp	20 bp	20 bp	20 bp	20 bp	20 bp	20 bp	20 bp	20 bp	20 bp	20 bp	20 bp	20 bp	20 bp	20 bp	20 bp	2 x 20 bp	2 x 20 bp	20 bp
CRISPR range [position]	74 to 93	74 to 93	74 to 93	74 to 93	74 to 93	C at pos 38, 48	C at pos 59, 61, 65, 66, 67	C at pos 64, 67, 71, 72, 73	64 to 83	64 to 83	57 to 76	57 to 76	57 to 76	57 to 76	64 to 83	64 to 83	8 to 27 108 to 127	8 to 27 108 to 127	10 to 29
PAM	GGG	GGG	GGG	GGG	GGG	CGG	TGG	CGG	CGG	CGA	CGC	CGG	CGC	CGG	CGG	CGA	CGG and AGG	CGG and AGG	AGG
Comments									sg RNA starts with C	sg RNA starts with C							Additional A in sgRNA 1	Additional A in sgRNA 1	
Input PEAR	12 882 586	12 831 242	14 423 628	12 190 759	13 240 911	13 893 148	15 279 496	14 649 301	13 083 171	13 083 171	12 907 732	12 907 732	16 723 719	16 723 719	17 372 664	17 372 664	16 887 348	16 672 816	10 233 949
Output PEAR	12 783 896	12 740 970	14 293 373	12 130 292	13 177 291	13 844 492	15 223 686	14 502 213	13 001 792	13 001 792	12 848 677	12 848 677	16 646 545	16 646 545	17 277 008	17 277 008	16 728 862	16 532 936	9 804 450
Output FASTQ-MCF	12 783 888	12 740 380	14 293 212	12 127 500	13 175 115	13 824 818	15 220 295	13 172 654	13 001 511	13 001 511	12 833 729	12 833 729	16 645 706	16 645 706	17 275 940	17 275 940	16 688 359	16 525 757	9 796 570
Sequence with N	4 874	4 804	5 625	1 093	1 171	1 234	1 363	844	980	980	928	928	6 149	6 149	6 823	6 823	5 817	5 803	74
Ratio with N/output FASTQ-MCF	0,00038	0,00038	0,00039	0,00009	0,00009	0,00009	0,00009	0,00006	0,00008	0,00008	0,00007	0,00007	0,00037	0,00037	0,00039	0,00039	0,00035	0,00035	0,00001
Input collapse	12 779 014	12 735 576	14 287 587	12 126 407	13 173 944	13 823 584	15 218 932	13 171 810	13 000 531	13 000 531	12 832 801	12 832 801	16 639 557	16 639 557	17 269 117	17 269 117	16 682 542	16 519 954	9 796 496
Unique sequences	2 181 556	2 347 896	2 350 731	584 410	518 172	348 632	429 856	597 698	303 287	303 287	370 305	370 305	2 977 395	2 977 395	2 103 634	2 103 634	3 631 475	4 271 539	1 607 901
NW below score	1 917	7 923	2 692	50 826	1 466	274	517	3 030 161	3 688	4 000	3 022	1 173	888 712	291 747	5 150	959	196 778	500 250	18 119
Unique after NW	2 179 639	2 339 973	2 348 039	533 584	516 706	348 358	429 339	NA	299 599	299 287	367 283	369 132	2 088 683	2 685 648	2 098 484	2 102 675	3 434 697	3 771 289	1 589 782
Ratio unique NW/input collapse	0,17	0,18	0,16	0,04	0,04	0,03	0,03	NA	0,02	0,02	0,03	0,03	0,13	0,16	0,12	0,12	0,21	0,23	0,16
Unique mutations	44 438	48 886	70 971	19 685	13 115	2 932	3 312	2 401	1 686	1 825	903	1 332	1 828	2 821	3 392	6 699	25 329	27 039	15 839
Mutation rate based on logo	High	High	High	High	High	High	High	Low	Medium	Low	Low	High	High	High	Medium	Medium	High	High	High
deletion-out	205 010	211 724	245 993	82 013	82 124	54 687	60 422	44 941	65 959	2 637	8 977	8 363	13 324	28 768	11 715	31 797	411 299	66 843	17 352
insertion-out	76 303	92 043	104 503	39 738	24 078	27 463	31 310	4 394	26 581	504	915	901	3 280	4 290	3 504	8 637	191 650	25 262	12 383
mismatch-out	46 318 014	45 584 505	54 356 829	13 108 835	15 260 072	13 769 598	15 582 025	6 651 894	6 220 271	1 494 552	2 940 192	2 967 750	20 890 526	22 342 650	16 430 243	45 324 132	68 043 647	46 527 826	18 376 283
deletion-in	2 693 543	3 604 848	1 023 569	3 099 745	2 764 839	4 870	6 735	5 405	23 895	305	5 344	401 880	31 400	136 243	37 106	218 354	11 659 007	11 700 693	2 458 735
insertion-in	319 492	431 294	583 424	598 147	381 864	157	448	1 101	11 242	57	865	65 397	1 947	12 326	12 411	80 407	751 817	1 282 959	1 339 969
mismatch-in	2 294 906	2 225 837	2 572 629	473 754	440 567	727 601	2 885 964	605 489	209 946	83 160	207 271	122 838	1 356 174	1 433 101	288 794	776 127	32 565 839	42 660 121	5 074 345
Target excluding primers	170	170	170	170	170	143	143	116	135	135	122	122	122	122	135	135	157	150	100
CRISPR range [length]	20	20	20	20	20	20	20	20	20	20	20	20	20	20	20	20	40	40	20
Target no primer no CRISPR	150	150	150	150	150	123	123	96	115	115	102	102	102	102	115	115	117	110	80
normalized deletion-out per base	1 367	1 411	1 640	547	547	445	491	468	574	23	88	82	131	282	102	276	3 515	608	217
normalized insertion-out per base	509	614	697	265	161	223	255	46	231	4	9	9	32	42	30	75	1 638	230	155
normalized mismatch-out per base	308 787	303 897	362 379	87 392	101 734	111 948	126 683	69 291	54 089	12 996	28 825	29 096	204 809	219 046	142 872	394 123	581 570	422 980	229 704
normalized deletion-in per base	134 677	180 242	51 178	154 987	138 242	244	337	270	1 195	15	267	20 094	1 570	6 812	1 855	10 918	291 475	292 517	122 937
normalized insertion-in per base	15 975	21 565	29 171	29 907	19 093	8	22	55	562	3	43	3 270	97	616	621	4 020	18 795	32 074	66 998
normalized mismatch-in per base	114 745	111 292	128 631	23 688	22 028	36 380	144 298	30 274	10 497	4 158	10 364	6 142	67 809	71 655	14 440	38 806	814 146	1 066 503	253 717
ratio deletion in/out	98,54	127,70	31,21	283,47	252,50	0,55	0,69	0,58	2,08	0,67	3,04	245,08	12,02	24,15	18,21	39,49	82,91	481,38	566,79
ratio insertion in/out	31,40	35,14	41,87	112,89	118,95	0,04	0,09	1,20	2,43	0,65	4,82	370,17	3,03	14,65	20,37	53,53	11,47	139,66	432,84
ratio mismatch in/out	0,37	0,37	0,35	0,27	0,22	0,32	1,14	0,44	0,19	0,32	0,36	0,21	0,33	0,33	0,10	0,10	1,40	2,52	1,10

Supplementary Table S6 | Mutations in *ZmSweet* genes in stable maize transformants

Mutation type	Mutation position	Frequency at sgRNA1 target in *ZmSWEET14a*	Frequency at sgRNA2 target in *ZmSWEET14a*	Frequency at sgRNA1 target in *ZmSWEET14b*	Frequency at sgRNA2 target in *ZmSWEET14b*	Frequency at sgRNA1 target in *ZmSWEET15a*
**Insertion**	1 bp at position -1	N.A	62,5 % (10/16)	100% (1/1)	20% (2/10)	N.A
	1 bp at position -2	N.A	N.A	N.A	10% (1/10)	N.A
**Deletion**	1 bp at position -1	N.A	18,75% (3/16)	N.A	N.A	N.A
	1 bp at position +1	N.A	N.A	N.A	10% (1/10)	N.A
	5 bp at position -2 to +3	N.A	N.A	N.A	20% (2/10)	N.A
	2 bp at position -4 to -3	N.A	6,25% (1/16)	N.A	N.A	N.A
	2 bp at position -2 to -1	N.A	6,25% (1/16)	N.A	N.A	N.A
	3 bp at position -3 to -1	N.A	6,25% (1/16)	N.A	10% (1/10)	N.A
	24 bp at position -19 to +5	N.A	N.A	N.A	10% (1/10)	N.A
	23 bp at position -9 to +14	N.A	N.A	N.A	N.A	100% (1/1)
	90 bp at position -84 to +6	N.A	N.A	N.A	20% (2/10)	N.A

Gene targeted ID Maize Genome V5	Allele	Sequence[1]	Position from canonical cut site	Occurrence
ZmSWEET14a Zm00001e011125	WT A188	GATCCTCCTCCTCCTCAACGTGGGCGTGTTCGGCCTCATCCTC-(50nt)-CTGGGTCTGCGTCGCCTTCT-CCGTCAGCGTCTTCGTCGCGCCGCT		
	ins T	GATCCTCCTCCTCCTCAACGTGGGCGTGTTCGGCCTCATCCTC-(50nt)-CTGGGTCTGCGTCGCCTTCTTCCGTCAGCGTCTTCGTCGCGCCGCT	-1	2
	ins A	GATCCTCCTCCTCCTCAACGTGGGCGTGTTCGGCCTCATCCTC-(50nt)-CTGGGTCTGCGTCGCCTTCTACCGTCAGCGTCTTCGTCGCGCCGCT	-1	5
	ins C	GATCCTCCTCCTCCTCAACGTGGGCGTGTTCGGCCTCATCCTC-(50nt)-CTGGGTCTGCGTCGCCTTCTCCCGTCAGCGTCTTCGTCGCGCCGCT	-1	3
	del C	GATCCTCCTCCTCCTCAACGTGGGCGTGTTCGGCCTCATCCTC-(50nt)-CTGGGTCTGCGTCGCCTTCT--CGTCAGCGTCTTCGTCGCGCCGCT	-1	3
	del CC	GATCCTCCTCCTCCTCAACGTGGGCGTGTTCGGCCTCATCCTC-(50nt)-CTGGGTCTGCGTCGCCTTCT---GTCAGCGTCTTCGTCGCGCCGCT	-1 to -2	1
	del CCG	GATCCTCCTCCTCCTCAACGTGGGCGTGTTCGGCCTCATCCTC-(50nt)-CTGGGTCTGCGTCGCCTTCT----TCAGCGTCTTCGTCGCGCCGCT	-1 to -3	1
	del GT	GATCCTCCTCCTCCTCAACGTGGGCGTGTTCGGCCTCATCCTC-(50nt)-CTGGGTCTGCGTCGCCTTCT-CC--CAGCGTCTTCGTCGCGCCGCT	-3 to -4	1
ZmSWEET14b Zm00001e021494	WT A188	GATCCTCCTCCTCCTCAACGTGGGCGT-GTTCGGGCTCATCCTC-(50nt)-CTGGGTCTGCGTCGCCTTCT-C-CGTCAGCGTCTTCGTCGCGCCGCT		
	ins T	GATCCTCCTCCTCCTCAACGTGGGCGTTGTTCGGGCTCATCCTC-(50nt)-CTGGGTCTGCGTCGCCTTCT-C-CGTCAGCGTCTTCGTCGCGCCGCT	-1	1
	ins A	GATCCTCCTCCTCCTCAACGTGGGCGT-GTTCGGGCTCATCCTC-(50nt)-CTGGGTCTGCGTCGCCTTCTAC-CGTCAGCGTCTTCGTCGCGCCGCT	-1	2
	ins T	GATCCTCCTCCTCCTCAACGTGGGCGT-GTTCGGGCTCATCCTC-(50nt)-CTGGGTCTGCGTCGCCTTCT-CTCGTCAGCGTCTTCGTCGCGCCGCT	-2	1
	del T	GATCCTCCTCCTCCTCAACGTGGGCGT-GTTCGGGCTCATCCTC-(50nt)-CTGGGTCTGCGTCGCCTTC--C-CGTCAGCGTCTTCGTCGCGCCGCT	+1	1
	del CCG	GATCCTCCTCCTCCTCAACGTGGGCGT-GTTCGGGCTCATCCTC-(50nt)-CTGGGTCTGCGTCGCCTTCT-----TCAGCGTCTTCGTCGCGCCGCT	-1 to -3	1
	del TCTCC	GATCCTCCTCCTCCTCAACGTGGGCGT-GTTCGGGCTCATCCTC-(50nt)-CTGGGTCTGCGTCGCCT-------GTCAGCGTCTTCGTCGCGCCGCT	-2 to +3	2
	del 24 bp	GATCCTCCTCCTCCTCAACGTGGGCGT-GTTCGGGCTCATCCTC-(50nt)-CTGGGTCTGCGTCGC--------------------------GCCGCT	-19 to +5	1
	del 90 bp	GATCCTCCTCCTCCTCAACGTGGGCGT-GTTCGGGCTCATCCTC-(50nt)-CTGGGTCTGCGTCG---------------------------------	-84 to +6	2
ZmSWEET15a Zm00001e022582	WT A188	CCTGGCCATGTACCTGGTGTACGCGCCCAAGGCCGCCCGGGTGCTGGCGGCC-(350nt)-ACGTGTTCGTGGCGTTCCCCAACGTGCTGGGCTTCGTCTTCGG		
	del 23 bp	CCTGGCCATGTACCTGG-----------------------GTGCTGGCGGCC-(350nt)-ACGTGTTCGTGGCGTTCCCCAACGTGCTGGGCTTCGTCTTCGG	-9 to +14	1

[1] The target sites are underlined, the PAM sites are in blue and the mutations in red
